# Prediction of blood-based biomarkers and subsequent design of bisulfite PCR-LDR-qPCR assay for breast cancer detection

**DOI:** 10.1186/s12885-020-6574-4

**Published:** 2020-01-31

**Authors:** Manny D. Bacolod, Jianmin Huang, Sarah F. Giardina, Philip B. Feinberg, Aashiq H. Mirza, Alexander Swistel, Steven A. Soper, Francis Barany

**Affiliations:** 1 Department of Microbiology and Immunology, Weill Cornell Medicine, New York, NY 10065 USA; 2 Department of Surgery, Weill Cornell Medicine, New York, NY 10065 USA; 3 Department of Mechanical Engineering, The University of Kansas, Lawrence, KS 66047 USA

**Keywords:** Breast cancer, Methylation, Early detection, Ligase detection reaction, Biomarker

## Abstract

**Background:**

Interrogation of site-specific CpG methylation in circulating tumor DNAs (ctDNAs) has been employed in a number of studies for early detection of breast cancer (BrCa). In many of these studies, the markers were identified based on known biology of BrCa progression, and interrogated using methyl-specific PCR (MSP), a technique involving bisulfite conversion, PCR, and qPCR.

**Methods:**

In this report, we are demonstrating the development of a novel assay (Multiplex Bisulfite PCR-LDR-qPCR) which can potentially offer improvements to MSP, by integrating additional steps such as ligase detection reaction (LDR), methylated CpG target enrichment, carryover protection (use of uracil DNA glycosylase), and minimization of primer-dimer formation (use of ribose primers and RNAseH2). The assay is designed to for breast cancer-specific CpG markers identified through integrated analyses of publicly available genome-wide methylation datasets for 31 types of primary tumors (including BrCa), as well as matching normal tissues, and peripheral blood.

**Results:**

Our results indicate that the PCR-LDR-qPCR assay is capable of detecting ~ 30 methylated copies of each of 3 BrCa-specific CpG markers, when mixed with excess amount unmethylated CpG markers (~ 3000 copies each), which is a reasonable approximation of BrCa ctDNA overwhelmed with peripheral blood cell-free DNA (cfDNA) when isolated from patient plasma. The bioinformatically-identified CpG markers are located in promoter regions of *NR5A2* and *PRKCB*, and a non-coding region of chromosome 1 (upstream of *EFNA3*). Additional bioinformatic analyses would reveal that these methylation markers are independent of patient race and age, and positively associated with signaling pathways associated with BrCa progression (such as those related to retinoid nuclear receptor, PTEN, p53, pRB, and p27).

**Conclusion:**

This report demonstrates the potential utilization of bisulfite PCR-LDR-qPCR assay, along with bioinformatically-driven biomarker discovery, in blood-based BrCa detection.

## Background

In 2019, the projected number of new cases of and deaths due to breast cancer (BrCa) in the United States, are 271,270, and 42,260 respectively [1]. Worldwide, the corresponding numbers (2018 estimate) are 2,088,849, and 626,679 respectively [2]. It is the second-leading cause of cancer death in women, one in 8 of whom will acquire the disease in her lifetime. Although genetic predisposition (i.e. BRCA1/2 mutations) is an important contributing factor (5–10%) [3, 4], most BrCa cases are those without clear genetic link (it may still be due to unknown genetic risk, thus considered familial). While Stage I cases have close to 100% 5-year survival rate, those diagnosed at Stage IV have a 5-year relative survival rate of only 22%, accounting for 6–10% of new BrCa cases and 20–30% all of recurrent disease [5]. The early detection of BrCa saves lives and reduces the morbidity associated with the aggressive treatments required for treating late-stage cancers. Nevertheless, the primary diagnostic screening method, mammography, has high rates of false positive and false negatives, can result in over-diagnosis,, uses harmful radiation, and is an uncomfortable process for patients [6, 7]. This necessitates the pursuit of molecular markers more indicative of a tumor’s biological characteristics translatable to a reliable, non-invasive diagnostic assay. Over the years, there have been numerous reports indicating that either blood serum, plasma, or whole blood can harbor molecular biomarkers indicative of a progressing BrCa [3, 8, 9]. These markers include: secreted proteins (e.g. CA15–3, trefoil factors 1, 2, and 3), auto-antibodies (e.g. antibodies against human endogenous retrovirus-K(HML-2) and heterogeneous nuclear ribonucleoprotein F), lipids (e.g. C16:1, C18:3, C18:2), and microRNAs (e.g. miR-21, miR-221, miR-145). In addition to the blood-based markers mentioned above, there is growing field exploring the use of DNA fragments released by cancer cells (referred to as circulating tumor DNAs or ctDNAs) into the patient’s bloodstream as an indicator of cancer [10, 11]. Previous studies proved that genomic signatures (e.g. mutation, copy number variation, CpG methylation) found in cancer tissues are largely concordant with those identified in ctDNAs [12–18]. Already marketed are early cancer diagnostic tests based on interrogating site-specific CpG hypermethylation in ctDNAs isolated from patient plasma. These include: a) Epi proColon, ColoVantage, Realtime mS9, all of which detect methylation in the *SEPT9* gene for colon cancer detection [19]; b) Epi proLung which detects methylation of *SHOX2* for lung cancer detection [20], and c) Colvera, which detects methylation at *BCAT1* and *IKZF1* for colon cancer recurrence [21].

There are important considerations in the development of methylation-based early detection assays for BrCa (or any other cancer type). Although the levels of plasma-derived cell free DNA (cfDNA) in serum from cancer patients are indeed abnormally high in early- to late-stage cancers [22–24], only a small percentage are ctDNAs (most cfDNAs are hematological in origin). Another important concern is the selection of appropriate markers. At the very least, the selected CpG sites should be highly methylated in breast primary tumors (PTs) and practically unmethylated in peripheral blood. However, for a marker to be highly specific to BrCa PTs, it needs to have very low levels of methylation in normal breast tissues, and many other tumor types. In this report, we demonstrate a new and more sensitive assay for methylated CpG detection (incorporating various steps including ligase detection reaction), and a comprehensive approach to biomarker discovery using integrated public genomic datasets.

### Methods

#### Public genomic datasets

Analyzed for this study are various publicly available genomic datasets (Additional file 1: Supplement 1) such as those released by the TCGA project (https://www.cancer.gov/about-nci/organization/ccg/research/structural-genomics/tcga) [25] and those deposited in the Gene Expression Omnibus (https://www.ncbi.nlm.nih.gov/geo/). The primarily Illumina 450 K methylation array-generated TCGA datasets were previously compiled (and processed) in the UCSC Cancer Genomics website (https://genome-cancer.ucsc.edu/) [26, 27]. The TCGA cohorts included in our analyses are: breast invasive carcinoma [BRCA], adrenocortical carcinoma [ACC], bladder urothelial carcinoma [BLCA], cervical squamous cell carcinoma and endocervical adenocarcinoma [CESC], cholangiocarcinoma [CHOL], colon adenocarcinoma [COAD], lymphoid neoplasm diffuse large b-cell lymphoma [DLBC], esophageal carcinoma [ESCA], glioblastoma multiforme [GBM], head and neck squamous cell carcinoma [HNSC], kidney chromophobe carcinoma [KICH], kidney renal clear cell carcinoma [KIRC], kidney renal papillary cell carcinoma [KIRP], brain lower grade glioma [LGG], liver hepatocellular carcinoma [LIHC], lung adenocarcinoma [LUAD], lung squamous cell carcinoma [LUSC], mesothelioma [MESO], pancreatic adenocarcinoma [PAAD], pheochromocytoma and paraganglioma [PCPG], prostate adenocarcinoma [PRAD], rectum adenocarcinoma [READ], sarcoma [SARC], skin cutaneous melanoma [SKCM], stomach adenocarcinoma [STAD], testicular germ cell tumors [TGCT], thymoma [THYM], thyroid carcinoma [THCA], uterine corpus endometrial carcinoma [UCEC], uterine carcinosarcoma [UCS], and uveal melanoma [UVM]. Also crucial to our biomarker identification is the integration of various GEO datasets such as: GSE65820 (ovarian cancer PTs and matching normals) [28], GSE46306 (normal tissues of the cervix) [29], GSE99553 (gastric mucosa), GSE74104 (testis) [30], GSE77871 (adrenal tissues), GSE51954 (dermis and epidermis) [31], GSE64509 (various brain tissues) [32], GSE42861 (peripheral blood) [33], and GSE59250 (various immune cells from healthy individuals) [34]. The methylation data for BrCa cell lines were extracted from the GEO datasets GSE57342 [35], GSE68379 [36], GSE78875 [37], and GSE94943.

#### Bioinformatic and statistical analyses

##### Programs and other tools

All statistical analyses (comparative statistics, normalization, correlation and regression analyses, multivariate analyses, hierarchical clustering) were performed using JMP Pro 11/ JMP Genomics software (SAS, Cary, NC), and Gene-E (Broad Institute, Cambridge, MA). Genomic sequence extraction and alignment were performed through the UCSC Genome Browser (https://genome.ucsc.edu/) [38]. The OligoAnalyzer Tool from IDT (https://www.idtdna.com/pages/tools/oligoanalyzer) aided in our primer designs.

##### Prediction of blood-based, breast cancer-specific methylation markers

The primary task was to identify CpG markers whose high degree of methylation in blood cfDNA may be indicative of primary BrCa, but (as much as possible) not of normal breast cells, peripheral blood, and many other cancer types. There are multiple approaches which can be employed to accomplish this task. Our approach started with simplifying each subgroup (e.g. PTs, solid normals) of a given cohort (e.g. TCGA-BRCA, TCGA-COADREAD), into arbitrarily defined metrics. In this case, the CpG marker **P** for a given cohort **C**, and cohort subset **S** was associated with specific statistical value **V**, such as %UM, %IM, %LM, %HM, %UM + %IM, and %LM + %HM, wherein **UM, IM, LM, HM** respectively refer to **U**n**M**ethylated (β_P_ ≤ 0.15; β = fraction of methylation at marker **P**), **I**ndeterminately **M**ethylated (0.15 < β_P_ ≤ 0.3), **L**owly **M**ethylated (0.3 < β_P_ ≤ 0.6), and **H**ighly **M**ethylated (β_P_ > 0.6) at the marker **P**. The candidate markers (**P’s**) were dynamically identified by isolating CpG sites which satisfied multiple criteria in the general form: **V**_**P**(**C,S**)_ ≥ n; {0 ≤ *n* ≤ 100}.

##### Assessment of methylation markers’ relationship with various clinico-pathological data

The accompanying clinico-pathological (such as pathological stage, PAM50 molecular subtype, age, race, and ethnicity) were used to assess their influence in the CpG markers’ β values.

##### Assessment of methylation markers’ roles in epigenetic regulation and other functionalities

The genome-wide transcriptional data (RNASeq-generated) for the BRCA cohort was also integrated into the analyses. First, the combined transcriptional and methylation data enabled us to predict if the CpG sites can potentially influence the transcription of their respective genes. Second, Gene Set Enrichment Analysis [39] was employed to predict the genes or pathways (http://software.broadinstitute.org/gsea/msigdb/genesets.jsp) that tend to be enriched (or upregulated) when a particular CpG site is highly methylated (such as some of the CpG sites which will eventually be verified experimentally).

#### Cell lines and genomic DNAs

The BrCa cell lines SKBr3, MDA-MB-134VI, and MCF7, which would serve as sources of cancer genomic DNAs (gDNAs), were grown according to culture conditions recommended by ATCC (https://www.atcc.org/). At 80–90% confluence, the cells were washed with Phosphate Buffered Saline (× 3), and collected by centrifugation (500 x g). gDNAs were isolated using the DNeasy Blood & Tissue Kit (Qiagen; Valencia, CA). gDNA (> 50 kb size) isolated from blood (buffy coat) of healthy individuals was purchased from Roche (Indianapolis, IN) (also referred to as “Roche DNA”). Quant-iT Picogreen Assay (Life Technologies/Thermo Fisher; Waltham, MA) was used to determine gDNA concentration. The isolated gDNAs were then fragmented (50 bp to 1 kb size) using an ultra-sonicator from Covaris (Woburn, Massachusetts). The fragmentation size was assessed using the Agilent Bioanalyzer System.

#### Enrichment of methylated genomic DNA

The gDNA fragments containing CpG methylated fragments were enriched using the EpiMark® Methylated DNA Enrichment Kit (New England BioLabs, Ipswich, MA). This approach uses selective binding of double-stranded methyl-CpG DNA to the methyl-CpG binding domain of human MBD2 protein fused to the Fc tail of human IgG1. The fused IgG1 (MBD2-Fc) antibody is coupled to paramagnetic hydrophilic protein A magnetic beads. The enrichment procedure was carried out according to the manufacturer’s instructions.

#### Bisulfite conversion of digested genomic DNA

Bisulfite conversion of cytosine bases was accomplished using the EZ DNA Methylation-Lightning kit from Zymo Research Corporation (Irvine, CA). In brief, 130 μl of Lightning Conversion Reagent was added to 20 μl of previously enriched gDNA fragments. Subsequent protocol steps (according to the manufacturer’s instructions) led to elution of bisulfite converted DNA fragments in 10 μl of elution buffer.

#### PCR-LDR-qPCR

The assay we developed for detection of plasma-based BrCa methylation markers is divided into several steps described in following subsections. All primers (Additional file 1: Supplement 2) were purchased from Integrated DNA Technologies Inc. (Coralville, IA).

#### Linear amplification

In a 25 μl of reaction volume, the linear amplification step was carried out by mixing: 5.0 μl of corresponding bisulfite converted DNA template (out of 50 μl of eluted DNA after bisulfite conversion), 5 μl of 5x GoTaq Flexi buffer (no Magnesium) (Promega, Madison, WI), 2.5 μl of 25 mM MgCl_2_ (Promega, Madison, WI), 0.5 μl of 10 mM dNTPs (dATP, dCTP, dGTP and dTTP) (Promega, Madison, WI), 2.5 μl of the reverse primer (or primers in case of multiplex reaction) (1 μM), 0.625 μl of 20 mU/μl RNAseH2 (diluted in RNAseH2 dilution buffer from IDT) (IDT), and 0.55 μl of KlenTaql polymerase (DNA Polymerase Technology, St. Louis, MO) mixed with Platinum Taq Antibody (Invitrogen/Thermo Fisher, Waltham, MA). The reactions were run in a ProFlex PCR system thermocycler (Applied Biosystems/ ThermoFisher, Waltham, MA) with the following program: 2 min at 94 °C, 40 cycles of (20 s at 94 °C, 40 s at 60 °C, and 30 s at 72 °C.), and a final hold at 4 °C. After the reaction, Platinum Taq antibodies were added in the reaction mixture to inhibit the KlenTaq DNA polymerase. The KlenTaql/Platinum Taq Antibody mixture was prepared by adding 0.02 μl of Klentaql polymerase at 50 U/μl to 0.2 μl of Platinum Taq Antibody at 5 U/μl.

#### PCR

For the PCR reaction, 10 μl of linear amplification product (previous step) was mixed with 2 μl of 5X GoTaq Flexi buffer without Magnesium, 1 μl of 25 mM MgCl_2_, 0.4 μl of dNTPs (10 mM each of dATP, dCTP, dGTP and dUTP), 2 μl of 0.5 μM forward primer (or primers in case of multiplex reaction), 0.4 μl of Antarctic Thermolabile UDG (1 U/μl) (New England Biolabs, Ipswich, MA), 0.25 μl of 20 mU/μl RNAseH2, 0.44 μl of KlenTaql polymerase mixed with Platinum Taq Antibody (Invitrogen/Thermo Fisher, Waltham, MA). The KlenTaql / Platinum Taq Antibody mixture was prepared by adding 0.02 μl of 50 U/μl Klentaql polymerase to 0.2 μl of 5 U/μl Platinum Taq Antibody. The 20 μl-volume reactions were run in a ProFlex PCR system thermocycler, using the following program: 10 min at 37 °C, 40 cycles of (20 s at 94 °C, 40 s at 60 °C. and 30 s at 72 °C), 10 min at 99.5 °C, and a final hold at 4 °C.

#### LDR

The LDR step was performed in a 20 μl reaction prepared by combining: 5.82 μl of nuclease-free water (IDT), 2 μl of 10X AK16D ligase reaction buffer 0.5 μl of 40 mM DTT (Sigma-Aldrich, St. Louis, MO), 0.25 μl of 40 mM NAD+ (Sigma-Aldrich, St. Louis, MO), 0.5 μl of 20 mU/μl RNAseH2, 0.4 μl of 500 nM LDR upstream probes, 0.4 μl of 500 nM LDR downstream probes, 0.57 μl of purified AK16D ligase (at 0.88 μM), and 4 μl of PCR reaction products from previous step. The AK16D ligase reaction buffer (at 1X) contains the following: 20 mM Tris-HCI at pH 8.5, 5 mM MgCl_2_, 50 mM KCl, 10 mM DTT, and 20 μg/ml of BSA (all components purchased from Sigma­ Aldrich, St. Louis, MO). LDR reactions were run in a ProFlex PCR system thermocycler using the following program: 20 cycles of (10 s at 94 °C, and 4 min at 60 °C) followed by a final hold at 4 °C.

#### Taqman real-time qPCR

The qPCR reaction was performed in a 10 μl of reaction mixture prepared by mixing: 1.5 μl of nuclease-free water (IDT), 5 μl of 2X TaqMan® Fast Universal PCR Master Mix (Fast AmpliTaq, UDG and dUTP)(Applied Biosystems/ThermoFisher; Waltham, MA), 1 μl 2.5 μM forward primer at, 1 μl of 2.5 μM reverse primer, 0.5 μl of 5 μM probe, and 1 μl of LDR reaction products from the previous step. All qPCR reactions were run in a ViiA7 real-time thermo-cycler from Applied Biosystems (Applied Biosystems/ThermoFisher; Waltham, MA), using MicroAmp® Fast-96-Well Reaction 0.1 ml plates sealed with MicroAmp™ Optical adhesive film (Applied Biosystems/ThermoFisher; Waltham, MA). The run settings were as follows: fast block, Standard curve as experiment type, ROX as passive reference, TAMRA as reporter, and NFQ-MGB as quencher; program at 2 min at 50 °C, and 40 cycles of (1 s at 95 °C, and 20 s at 60 °C).

#### Taqman digital qPCR

For each digital PCR reaction, a 20 μl mixture was prepared in each of the 96 well digital PCR microplate. The mixture included 2 μl of diluted LDR product (Step 3), 1X Luna Universal ProbeqPCR master mixture, 0.1% tween 20, 0.4 mU RNAseH2, 0.025 U Antarctic Thermolabile UDG, 5 μM each of forward and reverse primers, and Taqman probe. 12 μl of reaction mixture was loaded into the Constellation Digital PCR System (originally Formulatrix, Bedford, MA; currently Qiagen), and run with the following conditions: 37 °C for 10 min, 95 °C for 20 s, and 45 cycles of 5 s (94 °C), and 20 s (60 °C).

## Results

### Identification of potential blood-based breast cancer (BrCa) methylation markers

**Fig. 1:**
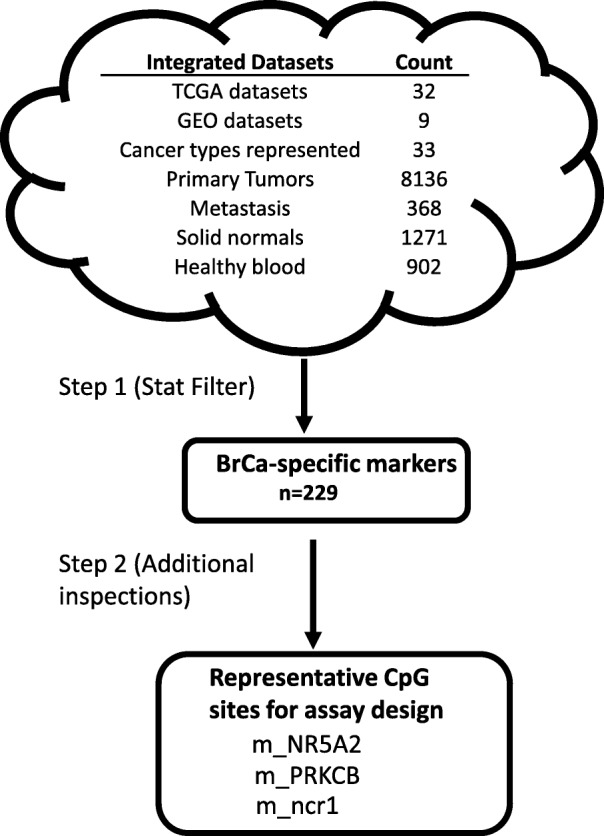
The scheme employed to identify potential site-specific methylation markers for blood-based early detection of breast cancer

**Fig. 2:**
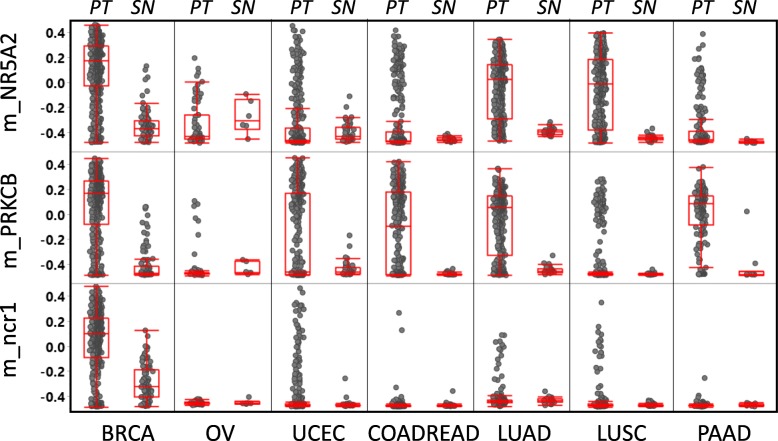
Comparative methylation levels (β values) of the 3 CpG sites (interrogated in the multiplex assay) in breast (BRCA) and other major cancer types among women: colorectal (COADREAD), ovarian (OV), endometrial (UCEC), lung (LUAD, LUSC), and pancreatic (PAAD). The β values range from − 0.5 (0%methyaltion) to + 0.5 (100% methylation)

The BrCa methylation markers were identified (See Fig. 1) by applying the following filters: **a**) %HM _(BRCA, PT)_ ≥ 30; **b**) %UM _(BRCA, SN)_ ≥ 40; **c**) Average (%UM) _(C, S)_ ≥ 40; C = any cancer cohort ≠ BRCA, S = PTs, **d**) Ave. (%UM) _(C, S)_ ≥ 40; C = any cancer cohort ≠ BRCA; S = Solid normals; and **e**) % UM _(GSE42861,blood)_ ≥ 98. A total of 229 CpG sites passed these filters. Among these are located in the loci of *CPXM1*, *RASSF1A*, and *SC3BGA1*, which have been reported in the literature to be indicative of BrCa [3]. Selected for further studies are the CpG sites located in the promoter regions of *NR5A2* (*nuclear receptor subfamily 5 group A member*) (referred to as “m_NR5A2) and *PRKCB* (*protein kinase C beta*)(m_PRKCB), as well as a CpG site in a non-coding region of chromosome 1 (m_ncr1) (Additional file 1: Supplement 3). The 3 aforementioned CpG sites were selected after further statistical inspections (i.e. the ones most likely to differentiate BrCa with other major cancer types), and assessment of surrounding sequences (i.e. to make sure assay can be designed around the target CpG sites). Of the 3 markers, m_ncr1 appears to be most highly specific to BrCa PTs (Fig. 2, Additional file 1: Supplement 4). The average methylation ($\overline{\upbeta}$) of m_ncr1, among BRCA PTs is 0.536 (on the scale 0 to 1, with 1 being 100% methylated). For other major cancer types such as ovarian cancer (OV), endometrial cancer (UCEC), colorectal cancer (COADREAD), lung adenocarcinoma (LUAD), lung squamous cell carcinoma (LUSC), and pancreatic cancer (PAAD), the $\overline{\upbeta}$ values for PTs (or $\overline{\upbeta}$_PT_) range from 0.030 to 0.093. In contrast, the $\overline{\upbeta}$ for normal tissues (or $\overline{\upbeta}$_SN_) is 0.206 for normal breast, and ranged from 0.028 to 0.066 in other tissue types. As shown in Fig. 2 and Additional file 1: Supplement 4, m_NR5A2 is very highly methylated in BRCA PTs ($\overline{\upbeta}$_PT_ = 0.604). However, it also exhibits a high level of methylation in lung cancer ($\overline{\upbeta}$_PT_ equals 0.454 and 0.436 for LUAD and LUSC PTs respectively). Among the major cancer types, the marker m_PRKCB exhibits the highest degree of methylation ($\overline{\upbeta}$_PT_ equals 0.561). Nonetheless, this particular marker may also test positive for LUAD ($\overline{\upbeta}$_PT_ = 0.446), PAAD ($\overline{\upbeta}$_PT_ = 0.498), UCEC ($\overline{\upbeta}$_PT_ = 0.328), and COADREAD ($\overline{\upbeta}$_PT_ = 0.362).

### Relationship of select CpG markers with other clinicopathological profiles

It is also possible to examine the various clinico-pathological information which may be associated with the methylation markers’ β values. As expected, more progressive tumors tend to have higher $\overline{\upbeta}$_PT_ values. For example, the m_ncr1 $\overline{\upbeta}$_PT_ values for Stage I, II, and III are 0.499, 0.534, and 0.590 respectively (Additional file 1: Supplement 5). For m_NR5A2, these respective values are 0.552, 0.610, and 0.662. For the m_PRKCB marker, while Stage III PTs registered the highest $\overline{\upbeta}$_PT_ (0.611), the $\overline{\upbeta}$_PT_ value for Stage I (0.562) is slightly higher than that of Stage II (0.548). Nonetheless, the most statistically significant difference, across all markers, is between Stage I and Stage III PTs. Another noteworthy observation is that in all 3 markers, the Basal subtype registered the lowest $\overline{\upbeta}$_PT_ values. For example, the m_ncr1 $\overline{\upbeta}$_PT_ values for Basal, Luminal A (LumA), Luminal B (LumB), and HER2 tumors are 0.440, 0.532, 0.628, and 0.630 respectively (Additional file 1: Supplement 6). We did not find a substantial correlation (R^2^ values range from 0.0 to 0.04) between patient age and methylation at each marker (irrespective of whether the tissue sample is a that of PT or solid normal) (See Additional file 1: Supplement 7). In addition, the methylation at each of the marker is independent of the racial (whether Asian, African American, or White) or ethnic (whether Hispanic or non-Hispanic) classification of the patient (Additional file 1: Supplements 8 and 9).

### The predicted biological roles of BrCa-specific methylation markers

**Fig. 3:**
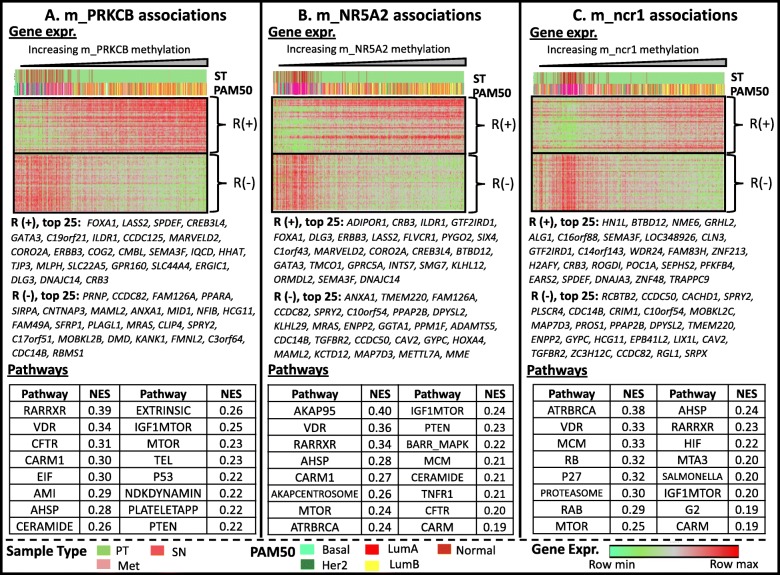
Heatmaps depicting the genes whose transcript levels in breast cancer samples are most highly correlated (negative or positive) with the methylation at the 3 select CpG sites: **a**) m_PRKCB, **b**) m_NR5A2, **c**) m_ncr1

The methylation level of either m_NR5A2 or m_PRKCB, apparently does not correlate with its transcript level (the respective R values are − 0.198 and − 0.123) (Additional file 1: Supplement 10). Nonetheless, it is still possible to bioinformatically demonstrate the association of these two CpG sites (along with m_ncr1, which is not part of the transcribed gene) with BrCa progression. It was accomplished in several ways. The first approach (See Fig. 3) was to identify the genes whose expression levels are most highly correlated with the methylation of the select markers. Indeed, a number of genes whose transcription positively and negatively correlated with each of the markers are known oncogenes and transcription factors respectively. For example, the expression of the transcription factor FOXA1, is highly (and positively) correlated with the methylation levels at m_PRKCB and m_NR5A2 (Fig. 3a and b). This transcription factor is needed for the transactivation of p27^Kip1^ [40], which alone or in conjunction with BRCA1 may control gene expression pattern in luminal subtypes of BrCa [41]. Similarly, the expression of SPDEF has a high positive correlation with m_PRKCB and m_ncr1(Fig. 3a and c). Previous studies indicated that SPDEF (also a transcription factor) is upregulated in all al subtypes of BrCa (except basal), and is especially associated with HR phenotype and nodal metastasis [42]. ERBB3 expression, which is positively correlated with methylation at m_PRKCB and m_NR5A2 (Fig. 3a and b), can form a heterodimer with ERBB2, and subsequently activates downstream signaling leading to cell cycle progression [43]. Another transcription factor, GATA3 is positively correlated with both m_PRKCB and m_NR5A2 (Fig. 3a and b). Upregulated in all BrCA subtypes (except basal), GATA3 is associated with ER positive BrCa (shown to regulate the expression of ER gene itself) [44]. In contrast, many of the genes whose transcript levels are negatively correlated with the methylation at the 3 CpG sites are known tumor suppressor genes. These include the genes *CDC14B* and *SPRY2*. CDC14B is a protein tyrosine phosphatase which can regulate RNA Pol II and repress cell cycle transcription [45]. SPRY2 acts as tumor suppressor in BrCa by inhibiting the Ras/Mitogen-Activated Protein Kinase Pathway [46], and in ovarian cancer, through inhibition of Amphiregulin (AREG)-induced cell invasion [47].

Another approach to bioinformatically justify the potential of methylation markers in cancer detection is through genome-wide transcriptional comparison between two BrCa PT subsets: those highly methylated (**H**), and those lowly methylated (**L**) at a particular CpG site p. The two subsets are defined as the follows: a) **H** subset includes PTs with β_p_ values higher by at least 1 σ from the mean ($\overline{\upbeta}$_p_), and b) **L** subset includes BrCa PTs with β_p_ values lower by at least 1 σ from the mean ($\overline{\upbeta}$_p_). Gene Set Enrichment Analysis (GSEA) [39] was then employed to identify the molecular pathways, as defined in Biocarta (https://www.gsea-msigdb.org/gsea/msigdb/genesets.jsp?collection=CP:BIOCARTA) or Reactome (https://reactome.org/) [48] databases, most likely enriched in **H** over **L** subsets. Results from this analysis would indicate that gene sets associated with retinoid nuclear receptor signaling, are highly enriched in **H** over **L** subsets. These include: RARRXR (*Nuclear receptors coordinate the activities of chromatin remodeling complexes and coactivators to facilitate initiation of transcription in carcinoma cells*), VDR (*Control of Gene Expression by Vitamin D Receptor*), and CARM1 (*Transcription Regulation by Methyltransferase of CARM1*). In the RARRXR pathway, the binding of retinoids to RARA/RXRA nuclear receptor heterodimer initiates the transcriptional activation of cell proliferation-associated phosphatidylinositol 3-kinase (PI3K) [49]. Other studies point to RARRXR pathway’s association with estrogen signaling [50, 51]. RARA/RXRA nuclear receptor plays a role in epigenetic regulation of Vitamin D Receptor (VDR). Previous studies have demonstrated VDR pathway’s association with BrCa proliferation [52]. The positive association between the methylation markers and CARM1 pathway is consistent with previous studies indicating that CARM1 (which codes for arginine methyltransferase) is involved in epigenetic transactivation of many nuclear receptors (NRs) including ERα [53]. CARM1 is also associated with poor prognosis in BrCa [54].

Also positively associated with at least one of the 3 methylation markers is the upregulation of cancer proliferation signaling pathways including: PTEN (*PTEN dependent cell cycle arrest and apoptosis*), P53 (*p53 Signaling Pathway*), P27 (*Regulation of p27 Phosphorylation during Cell Cycle Progression*), RB (RB Tumor Suppressor/Checkpoint Signaling in response to DNA damage), ATRBRCA (*Role of BRCA1, BRCA2 and ATR in Cancer Susceptibility*), and MTOR (*mTOR Signaling Pathway*). The enrichment of the aforementioned cancer pathways (in **H** over **L** PT subsets) may be explained by the upregulated expression of genes comprising these gene sets. These genes include: a) *MAPK3*, *PDK2*, and *PTEN*, of the PTEN gene set; b) *BCL2*, *CCND*1, *RB1*, *TIMP3*, and *MDM2* of the P53 gene set; c) *E2F1* and *CKS1B*, of the P27 gene set; d) *CDC25C*, *MYT1*, *CDK1*, and *CD25B* of the RB gene set; e) *RAD51*, *BRCA1*, *FANCD2*, and *BRCA2*, of the ATRBRCA gene set; and f) *EIF4EBP1* of the MTOR gene set.

### Multiplex bisulfite PCR-LDR-qPCR assay to validate the 3 breast cancer methylation markers

**Fig. 4:**
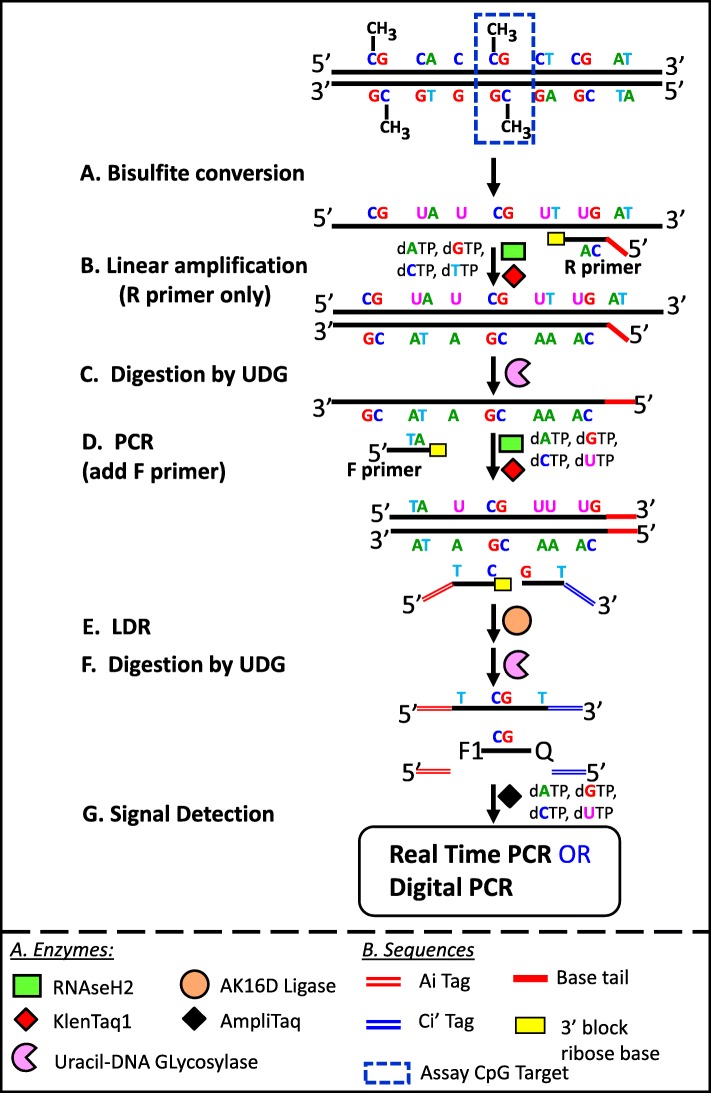
Schematic of the Bisulfite PCR-LDR-qPCR assay for detection of CpG methylation

**Fig. 5:**
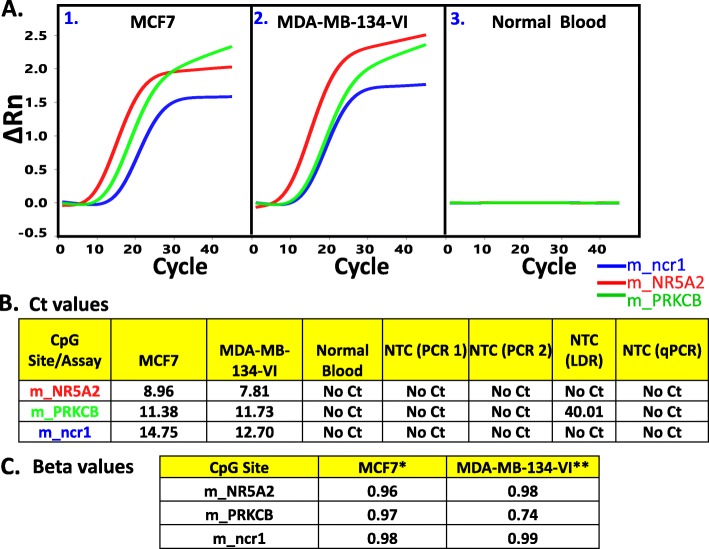
**a.** Panels **1** and **2** refer to the resulting Ct plots (from ViiA7 run) for multiplex detection of the CpG markers m_ncr1, m_NR5A2, and m_PRKCB using as initial template fragmented and bisulfite-converted mixture of 30, and 3000 genomic copies of DNA from breast cancer cell line (MCF7 or MDA-MB-134-VI) and normal human blood (Roche human genomic DNA) respectively. The DNA fragment mixture simulates the likely constitution of patient cfDNA (i.e. majority of which are released by peripheral blood cells). Panel **3** serves as a negative control (3000 copies of genomic DNA from normal human blood). **b**. The Ct values for the plots depicted in **A**. Also shown are results from no template controls (NTCs) in various steps of the assay (PCR, LDR, qPCR). “No Ct” means no amplification was detected after 45 cycles of real time PCR. **c**. The fraction of methylation at a specific CpG site for the 3 CpG sites in the genomes of MCF7 and MDA-MB-134-VI cell lines, as extracted from Illumina 450 K array-generated datasets deposited in Gene Expression Omnibus (GEO). * Average values extracted from datasets GSE57342, GSE68379, GSE78875, and GSE94943. **Value extracted from dataset GSE68379

In essence, the analyses described above rationalized that the methylation level at 3 CpG sites (m_ncR1, mNR5A2, and m_PRKCB) can serve as serum markers for progressing BrCa. With the aim of translating these findings to clinical use, we tested a homegrown procedure (bisulfite PCR-LDR-qPCR assay) (Fig. 4) designed to interrogate these methylated markers present in minuscule amount (i.e. down to less than 50 copies), as it is expected in cfDNA isolated from ~ 10-ml patient blood. The assay was tested on simulated cfDNA sample consisting of a mixture of gDNA fragments from BrCa cell line and peripheral blood from healthy patients, with the fragments from the latter present at ~ 100-fold relative to the former. It is a fair assumption that many of the BrCa cell lines will be highly methylated at the 3 CpG sites. Nonetheless, given the availability of genome-wide methylation datasets for many of the commonly used cancer cell lines, this assumption can easily be verified. According to the datasets GSE57342, GSE68379, GSE78875, and GSE94943, the average β values for the CpG markers m_NR5A2, m_PRKCB, and m_ncr1 in the BrCa cell line MCF7 are 0.96, 0.97, and 0.98 respectively (Fig. 5). For the cell line MDA-MB-134-VI, the respective numbers are 0.98, 0.74, and 0.99. With this information, the gDNAs from these cell lines were isolated, fragmented, and enriched for methylation (through the use of fusion protein with the methyl-CpG binding domain, which can selectively bind DNA fragments containing methylated CpG sites). As shown in the scheme (Fig. 4), the simulated cfDNA (~ 30 and 3000 copies of cell line and peripheral DNA fragments respectively), was bisulfite-converted, then subjected to two-step PCR. The resulting amplicon would then serve as template for ligase detection reaction (LDR). The final step to detect the methylation signal was Taqman-based real time PCR, using the LDR products (from the previous step) as templates. The Ct values for m_NR5A2, m_PRKCB, and m_ncr1 were 8.96, 11.38, and 14.75 respectively for MCF7, and 7.81, 11.73, 12.70 respectively for MDA-MB-134-VI. No Ct values registered for the unmixed peripheral blood for each of the marker. Indeed, the designed assays were capable of detecting minute amount of the BrCa methylation markers. These assays were designed to reduce false positive signals by minimizing non-specific amplifications Among the important features are the 8–11 base tails in the reverse PCR primers, meant to reduce the possibility of primer dimer formation. Also present were the ribose bases at the 3′ end of the PCR and LDR primers. These sequences were removed by RNaseH2 when the primers were bound to their targets, ensuring highly specific extension (for PCR) and ligation (for LDR). Carryover prevention was the objective of the timely addition of UDG, dUTP, or dTTP in the dNTP mixture [55]. Also, the tags in LDR primers (Ai, Ci’) allowed for uniformity (in terms of Tm) in qPCR reactions.

### Digital PCR as the final detection step

**Fig. 6:**
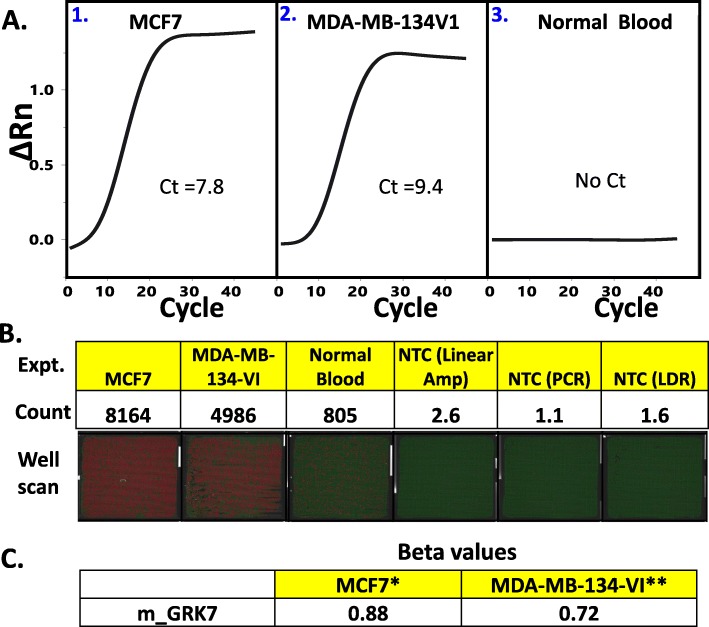
**a.** Panels **1** and **2** refer to the resulting Ct plots (from ViiA7 run) for multiplex detection of the CpG marker m_GRK7, using as template 30 copies of bisulfite-converted and fragmented genomic DNA from breast cancer cell line (MCF7 or MDA-MB-134-VI) mixed with 3000 copies of genomic DNA from human blood (Roche human genomic DNA). Panel **3** refers to negative control, with just the normal blood genomic DNA as a template. **b.** A digital PCR readout (using Formulatrix Constellation dPCR System) for similar experiments depicted in **A**. NTC refers to “No template control”. **c**. The fraction of methylation at a specific CpG site for m_GRK7 CpG site in the genomes of MCF7 and MDA-MB-134-VI cell lines. This information was extracted from Illumina 450 K array-generated datasets deposited in Gene Expression Omnibus (GEO). * Average value extracted from datasets GSE57342, GSE68379, GSE78875, and GSE94943. **Value extracted from dataset GSE68379

Instead of Taqman PCR, digital PCR may also be used as detection step. For this experiment, we performed the 2-step PCR and LDR reactions as described above, using the primers for the detection of methylation at the CpG site located in the promoter region of the gene *GRK7* (m_GRK7 or cg18768784; Chr3:_141516271–141,516,272), although highly methylated in the BrCa cohort, has low BRCA-specificity. Nonetheless, there is a high degree of concordance between detection based in qPCR (Fig. 6a) and detection using the Constellation (Formulatrix) digital PCR System (Fig. 6b). The bisulfite converted DNA fragments consisting of ~ 3000 copies of unmethylated m_GRK7 (from peripheral blood) and ~ 30 copies of methylated m_GRK7 yielded Ct values of 7.8 and 9.4 for MCF7 and MDA-MB-134VI respectively. The unmixed template (DNA fragments from peripheral blood only) did not register readable Ct. In the digital PCR detection system, the corresponding readings are 8164, 4986, and 805 for the MCF7, MDA-MB-134VI, and control (peripheral blood only) respectively.

## Discussion

The limitations of mammography are what drives the persistent efforts towards developing non-invasive screening approaches for early BrCa detection. Falling under the term “liquid biopsy”, many of the methods under investigation are technologies which aim to detect blood-based molecular markers originating from BrCa cells. The molecular markers can include cfDNA fragments, exosome-enclosed or naked RNA molecules, secreted proteins and metabolites [8, 9].

Of particular interest in the early-cancer detection field are circulating tumor DNAs (ctDNAs), which apoptotic and necrotic cancer cells release into patient plasma [56]. As expected, ctDNA fragments possess the same molecular signatures (somatic mutations, methylation, copy number variation/aberration, SNPs) present in gDNAs isolated from the tumor tissue samples. Hence, molecular characterization tools normally used to investigate cancer gDNAs (such as exome or genome-wide sequencing, PCR, DNA arrays, methylation arrays) have also been applied in ctDNA analysis [57]. What makes ctDNA analysis especially challenging is the fact that when isolated from patient plasma, ctDNAs are mixed with an overwhelming amount of DNA fragments that are hematopoietic in origin [58–60]. All of the fragments are collectively referred to as cell-free DNAs (or cfDNAs). According to a recent study, the ctDNAs originating from BrCa cells is just a small fraction of total cfDNAs [24]. This is based on the observation that the mutant allele fraction (MAF; from sequencing 58 cancer-related genes) of cfDNAs isolated from BrCa patients is less than 1% [24]. It is imperative that the assay employed to analyze cfDNAs is capable of distinguishing between the positive (several copies of ctDNAs) and mostly negative (from non-cancer cfDNAs) signals. This limitation of ctDNA analyses can be circumvented through the identification of more appropriate molecular biomarkers, along with the modification of assay biochemistry towards higher sensitivity and specificity. Although plasma-based ctDNA markers may include markers for mutations, methylation states, or copy number variations (most reports interrogate methylation and mutation markers), methylation markers have several inherent advantages. First, methylation changes are tissue-specific [61], thus as markers, would make them highly capable of distinguishing one cancer type from another. Another advantage of CpG methylation over mutation is that oftentimes the methylation changes adjacent CpG sites in promoter regions, are concordant. Methylation-dependent procedures (such as the use of methyl DNA-binding antibodies) would then be more effective in enriching the fragments containing the highly methylated markers.

Identification of appropriate methylation markers (i.e. particular CpG sites) is very crucial. To pinpoint the specific CpG sites that can easily distinguish BrCa tissues from peripheral blood and other types of cancer, we took advantage of the availability of various genome-wide methylation datasets. As previously pointed out, these calculations resulted in identification of 229 potential CpG markers which included CpG sites at the locus of *RASSF1A* (*Ras association domain-containing protein 1*), which happens to be the most highly reported blood-based methylation markers for breast cancer [62–69]. Additional statistical inspections and assay design considerations would then point to the selection of the 3 CpG markers we focused on for this manuscript. Two of the CpG sites (m_NR5A2 and m_PRKCB) are located in promoter regions of genes, with reported link to breast cancer. NR5A2 (or LRH1) is a zinc finger transcription factor which can regulate CDKN1A expression in BrCa [70], and has been positively associated with BrCa proliferation [71], drug resistance [72], aggressiveness [73], high grade, and poor outcome [74]. On the other hand, the role of PRKCB in breast cancer progression is still not clearly defined. While there are reports indicating that PRKCB can promote mammary tumorigenesis [75], enhance breast cancer cells growth and cyclin D1expression [76], and has the potential as therapeutic target [77], there is also a report indicating it may inhibit tumor growth and metastasis [78]. The third CpG site interrogated by our assay (m_ncr1) is located less than 8000 bp upstream of the exon 1 (according to GENCODE v31 annotation) of the protein coding gene *EFNA3*, a member of the ephrin (EPH) family. Whether this particular CpG site influences the expression of EFNA3 protein, or the hypoxia-related EFNA3 lncRNA [79, 80] is not clear at this point.

Interestingly, the methylation level at m_NR5A2 and m_PRKCB did not correlate with the transcription of the corresponding genes (Additional file 1: Supplement 10). However, it is important to note that CpG methylation (at the promoter region) is not the only factor that influences gene transcription. It is quite possible that histone modification [81], regulatory miRNA or ncRNAs [82], as well as transcription factors can supersede CpG methylation in influencing transcription. The competition between mRNA transcription and mRNA degradation is a dynamic process that can determine the transcript level of a gene at any given time [83]. Regardless of m_NR5A2 and m_PRKCB CpG sites’ influence on their respective transcript levels, their association with BrCa progression is quite clear. This is further demonstrated through comparative genome-wide transcription analyses (which is essentially what GSEA is) of BrCa samples that are highly and lowly methylated at each CpG site. As shown in our analyses, the methylation level at each of the three methylation markers (m_ncr1, m_NR5A2, and m_PRKCB), is positively associated with genes, processes, and pathways indicative of BrCa progression. These include processes (and much of the component genes) associated with the retinoid nuclear receptor, PTEN, p53, p27, RB, and MTOR signaling pathways.

A great majority of reports on the interrogation of CpG methylation in cfDNA for BrCa detection employed the methylation-specific PCR (MSP) approach. Aside from *RASSF1*, other genes whose CpG sites were observed to be hypermethylated in BrCa patient-derived cfDNAs (through MSP approach) are: *AKR1B*1, *ARHGEF7*, *BRCA1*, *BRMS1*, *COL6A2*, *CST6*, *CDKN2A*, *CCND2*, *DKK3*, *ESR1*, *GATA3*, *GPX7*, *GSTP1*, *HOXD13*, *HIST1H3C*, *HOXB4*, *ITIH5*, *KLK10*, *MSH2*, *MLH1*, *NBPF1*, *P16*, *PCDHGB7*, *RARB*, *RASGRF2*, *SOX17*, *SLIT2*, *SFN*, *SFRP1*, *SOX17*, *TM6SF1*, *TMEFF2*, *TRIM9*, and *WNT5A* [84] [62, 64–69, 85–92]. The aforementioned CpG markers were selected because the genes have known roles in BrCa progression (primarily as tumor suppressors), or were previously identified from the use of earlier, much less dense version of Illumina methylation array (27 K).

Bisulfite conversion is perhaps the most crucial step in MSP. However, bisulfite conversion can cause the degradation of around 84–96% of the input cfDNA, and is thus a significant contributing factor to MSP’s limitations in liquid biopsy [93]. This is not an issue in analyzing gDNAs extracted from tissues and cell lines, which the MSP assay was originally intended for. In some reports, BrCa patient cfDNAs were analyzed through methylated CpG digestion (e.g. BstUI enzyme), followed by qPCR, with no bisulfite conversion step in the protocols [94–96]. However, results using this approach are not reliable (higher rates of false positives) if there is incomplete digestion of unmethylated CpG sites.

The assay we are proposing incorporated several features which can collectively improve the MSP approach. These include the following: a) selective enrichment of methylated DNA, through the methylated CpG capture by using methyl-DNA binding protein, b) signal amplification of the targeted CpG site through successive steps of bisulfite PCR and LDR, (c) prevention of non-specific primer extension by incorporating RNaseH2-targeted ribose bases at the 3′ end of PCR and LDR primers, d) prevention of carryover-contamination by PCR products originating from previous positive samples, through the use UDG enzyme, e) multiple primer binding regions for orthogonal amplification of a region containing the targeted CpG site, and f) multiplex format of the assays.

Bisulfite sequencing is capable of interrogating more CpG markers compared to site-specific bisulfite conversion assays [97–102]. However, we can only assume that the primary problems in MSP assays (the low abundance of cfDNAs and of target methylated CpG markers) are also encountered in bisulfite sequencing approaches. These factors, along with high cost, limits the recovery of information from bisulfite sequencing of cfDNA fragments [103].

## Conclusion

This report demonstrates the steps which can be utilized to improve blood-based early detection assays for BrCa detection, including bioinformatic identification and characterization of the biomarkers and improvements in assay biochemistry. Understandably, the assays were only tested on simulated cfDNA samples. However, in future studies, we will evaluate the non-invasive BrCa diagnostic capability of our multiplex PCR-LDR-qPCR assay through analysis of patient-derived cfDNAs.

## Supplementary information

**Additional file 1: Supplement 1.** The cohorts analyzed for biomarker discovery. Legends: PT (primary tumors), SN (solid normal), PB (peripheral blood), Met (metastasis), Imm (immune cells). **Supplement 2**. List of primers used for the multiplex PCR-LDR-qPCR assay. **Supplement 3.** Genomic information for the breast cancer-specific CpG sites interrogated in the multiplex PCR, LDR, qPCR assay described in this report. **Supplement 4. A.** Statistical summary of the β values (methylation level) for 3 CpG markers across major types of cancer. β value ranges from 0 (ummethylated) to 1 (completely methylated). PT = primary tumor; SN = solid normal. **B-D**. Statistical comparison (for each of the marker) between each subgroup. **Supplement 5.** Relationship between CpG methylation and Primary Tumor Stage. **Supplement 6.** Relationship between CpG methylation and molecular subtypes of breast cancer. **Supplement 7.** Relationship between CpG methylation and patient age. **Supplement 8.** Relationship between CpG methylation and patient race. **Supplement 9** Relationship between CpG methylation and patient ethnicity. **Supplement 10.** Relationship between CpG methylation and gene expression.

## Data Availability

We did not generate new datasets for this article.

## Abbreviations

BrCa: Breast cancer
cfDNA: Cell-free DNA
ctDNA: Circulating tumor DNA
gDNA: Genomic DNA
LDR: Ligase detection reaction
PT: Primary tumor
