# Supplementary material for: Prediction of blood-based biomarkers and subsequent design of bisulfite PCR-LDR-qPCR assay for breast cancer detection
Source: BMC Cancer. 2020 Jan 31;20:85. doi: 10.1186/s12885-020-6574-4 (PMC6995062; doi:10.1186/s12885-020-6574-4)
Supplement: Supplementary file 1 — Additional file 1: Supplement 1. The cohorts analyzed for biomarker discovery. Legends: PT (primary tumors), SN (solid normal), PB (peripheral blood), Met (metastasis), Imm (immune cells). Supplement 2. List of primers used for the multiplex PCR-LDR-qPCR assay. Supplement 3. Genomic information for the breast cancer-specific CpG sites interrogated in the multiplex PCR, LDR, qPCR assay described in this report. Supplement 4. A. Statistical summary of the β values (methylation level) for 3 CpG markers across major types of cancer. β value ranges from 0 (ummethylated) to 1 (completely methylated). PT = primary tumor; SN = solid normal. B-D. Statistical comparison (for each of the marker) between each subgroup. Supplement 5. Relationship between CpG methylation and Primary Tumor Stage. Supplement 6. Relationship between CpG methylation and molecular subtypes of breast cancer. Supplement 7. Relationship between CpG methylation and patient age. Supplement 8. Relationship between CpG methylation and patient race. Supplement 9 Relationship between CpG methylation and patient ethnicity. Supplement 10. Relationship between CpG methylation and gene expression. [file 12885_2020_6574_MOESM1_ESM.pdf]

Supplementary Files

Supplement 1 (Table). The cohorts analyzed for biomarker discovery. Legends: PT (primary tumors), SN (solid normal), PB (peripheral blood), Met (metastasis), Imm (immune cells).

Cohort ID	Source	Cohort Description	Sample Count	Average Age	Primary Tumor Stage Distribution
ACC	TCGA	adrenocortical carcinoma	PT(80)	PT(46.4 ± 15.89)	I(9), II(36), III(16), IV(16)
BLCA	TCGA	bladder urothelial carcinoma	PT(412), SN(21)	PT(69.89 ± 12.56)	I(3), II(114), III(120), IV(118)
BRCA	TCGA	breast invasive carcinoma	PT(668), SN(98)	PT(57.77 ± 13.09); SN(57.42 ± 15.19)	I(50), IA(54), IB(5), II(8), IIA(205), IIB(148), III(2), IIIA(106), IIIB(17), IIIC(41), IV(6), X(5)
CESC	TCGA	cervical squamous cell carcinoma and endocervical adenocarcinoma	PT(307)	PT(48.23 ± 13.8)	I(5), IA(1), IA1(1), IA2(1), IB(37), IB1(77), IB2(38), II(5), IIA(9), IIA1(5), IIA2(7), IIB(43), III(1), IIIA(3), IIIB(42), IVA(9), IVB(12)
CHOL	TCGA	cholangiocarcinoma	PT(36), SN(9)	PT(63.34 ± 12.9); SN(70.56 ± 9.15)	I(19), II(9), III(1), IV(1), IVA(2), IVB(3)
COAD	TCGA	colon adenocarcinoma	PT(240), SN(38)	PT(65 ± 13.19); SN(69.37 ± 12.44)	I(43), IA(1), II(14), IIA(92), IIB(5), IIC(1), III(7), IIIA(9), IIIB(45), IIIC(23), IV(21), IVA(17), IVB(2)
DLBC	TCGA	lymphoid neoplasm diffuse large b-cell lymphoma	PT(48)	PT(56.17 ± 14.23)	I(8), II(17), III(4), IV(12)
ESCA	TCGA	esophageal carcinoma	PT(185), SN(16)	PT(62.62 ± 12.16); SN(67.69 ± 10.98)	0(1), I(8), IA(5), IB(7), II(1), IIA(39), IIB(29), III(26), IIIA(12), IIIB(10), IIIC(7), IV(4), IVA(3)
GBM	TCGA	glioblastoma multiforme	PT(140)	PT(59.75 ± 12.83)	NA
GSE42861	GEO	peripheral blood control	PB(689)	SN(51.92 ± 11.79)	NA

Cohort ID	Source	Cohort Description	Sample Count	Average Age	Primary Tumor Stage Distribution
GSE46306	GEO	cervical tissue	SN(20)	NA	NA
GSE51954	GEO	human dermis and epidermis	SN(79)	SN(50.25 ± 25.2)	NA
GSE59250	GEO	various immune cells	Imm(206)	NA	NA
GSE64509	GEO	human brain tissues	SN(260)	SN(71.2 ± 27.35)	NA
GSE65820	GEO	ovarian cancer	PT(79), SN(6)	NA	NA
GSE74104	GEO	testicular germ cell tumors	PT(139), SN(128)	NA	NA
GSE77871	GEO	adrenocortical cancer	PT(18), SN(6)	NA	NA
GSE99553	GEO	gastric cancer	PT(42), SN(42)	NA	NA
HNSC	TCGA	head and neck squamous cell carcinoma	PT(528), SN(50)	PT(60.93 ± 11.97); SN(62.06 ± 10.63)	I(27), II(78), III(75), IVA(253), IVB(12), IVC(1)
KICH	TCGA	kidney chromophobe carcinoma	PT(66)	PT(51.52 ± 14.3)	I(21), II(25), III(14), IV(6)
KIRC	TCGA	kidney renal clear cell carcinoma	PT(319), SN(160)	PT(61.52 ± 11.85); SN(63.13 ± 11.45)	I(152), II(31), III(75), IV(56)
KIRP	TCGA	kidney renal papillary cell carcinoma	PT(275), SN(45)	PT(61.36 ± 12.27); SN(63.78 ± 12.61)	I(163), II(18), III(48), IV(13)
LAML	TCGA	AML_PB	Primary cancer (194)	PT(55.11 ± 16.02)	NA
LGG	TCGA	lower grade glioma	PT(516)	PT(43.05 ± 13.51)	NA
LIHC	TCGA	liver hepatocellular carcinoma	PT(377), SN(50)	PT(59.81 ± 13.11); SN(62.34 ± 15.98)	I(168), II(83), III(3), IIIA(62), IIIB(8), IIIC(9), IV(3), IVA(1), IVB(2)
LUAD	TCGA	lung adenocarcinoma	PT(450), SN(32)	PT(65.11 ± 10.16); SN(64.56 ± 11.56)	I(6), IA(118), IB(125), II(1), IIA(47), IIB(64), IIIA(64), IIIB(9), IV(21)
LUSC	TCGA	lung squamous cell carcinoma	PT(372), SN(43)	PT(67.71 ± 8.65); SN(68.62 ± 10.02)	I(4), IA(69), IB(98), II(2), IIA(58), IIB(67), III(3), IIIA(46), IIIB(7), IV(4)
MESO	TCGA	mesothelioma	PT(87)	PT(62.89 ± 9.55)	I(6), IA(2), IB(1), II(15), III(39), IV(10
PAAD	TCGA	pancreatic adenocarcinoma	PT(184), SN(10)	PT(64.91 ± 11.16); SN(70.38 ± 13.44)	I(1), IA(6), IB(13), IIA(26), IIB(114), III(5), IV(4)

Cohort ID	Source	Cohort Description	Sample Count	Average Age	Primary Tumor Stage Distribution
PCPG	TCGA	Pheochromocytoma and paraganglioma_PT	PT(179)	PT(47.33 ± 15.12)	NA
PRAD	TCGA	prostate adenocarcinoma	PT(192), SN(49)	PT(60.87 ± 6.87); SN(61.88 ± 6.53)	NA
READ	TCGA	rectum adenocarcinoma	PT(91)	PT(62.94 ± 12.29); SN(66.57 ± 12.73)	I(11), II(6), IIA(20), IIB(2), IIC(2), III(4), IIIA(9), IIIB(13), IIIC(11), IV(6), IVA(9)
SARC	TCGA	sarcoma	PT(261)	PT(61.19 ± 14.41)	NA
SKCM	TCGA	skin cutaneous melanoma	Met(368), PT(105)	PT(63.39 ± 14.2); Met(55.80 ± 15.74)	IB(1), II(1), IIA(3), IIB(7), IIC(41), III(1), IIIA(1), IIIB(11), IIIC(12), IV(3),Mets(325)
STAD	TCGA	stomach adenocarcinoma	PT(260)	PT(65.22 ± 10.6)	I(3), IA(13), IB(36), II(28), IIA(39), IIB(54), III(3), IIIA(78), IIIB(60), IIIC(40), IV(34)
TGCT	TCGA	testicular germ cell tumors	PT(150)	PT(32.08 ± 9.37)	I(18), IA(24), IB(11), II(5), IIA(6), IIB(1), IIC(1), III(2), IIIA(1), IIIB(6), IIIC(5), IS(46)
THCA	TCGA	thyroid carcinoma	PT(500), SN(56)	PT(47.07 ± 15.56); SN(45.66 ± 16.87)	THCA, I(272), II(51), III(108), IV(2), IVA(41), IVC(5)
THYM	TCGA	Thymus_PT	PT(124)	PT(58.65 ± 12.61)	NA
UCEC	TCGA	uterine corpus endometrial carcinoma	PT(431), SN(46)	PT(64.22 ± 11.26); SN(60.97 ± 11.32)	I(2), IA(119), IB(114), IC(20), II(27), IIA(4), IIB(11), III(2), IIIA(35), IIIB(5), IIIC(24), IIIC1(16), IIIC2(16), IV(4), IVA(2), IVB(18)
UCS	TCGA	uterine carcinosarcoma	PT(57)	PT(69.74 ± 9.3)	IA(12), IB(8), IC(2), II(3), IIA(1), IIB(1), III(2), IIIA(2), IIIB(2), IIIC(4), IIIC1(4), IIIC2(6), IV(1), IVB(9)

Cohort ID	Source	Cohort Description	Sample Count	Average Age	Primary Tumor Stage Distribution
UVM	TCGA	uveal melanoma	PT(80)	PT(61.77 ± 14.03)	IIA(12), IIB(27), IIIA(24), IIIB(10), IIIC(1), IV(4)

Supplement 2 (Table). List of primers used for the multiplex PCR-LDR-qPCR assay. Note that: /5SpC3/ - 5' C3 Spacer, /3SpC3/ - 3' C3 Spacer, /5Phos/ - 5' Phosphate, /56-FAM/ - 5' Fam Flourescent Dye, /5HEX/ - HEX™ Fluorescent Dye, /ZEN/ - ZEN™ Flourescent Quencher ™, /3IABkFQ/ - 3' Iowa Black ® Flourescent Quencher , green to pink spectral range, "+" - Locked Nucleic Acid base, "rA" - ribonucleotide base riboadenosine; "rT" - ribonucleotide base ribothymidine; "rG" - ribonucleotide base riboguanosine; "rC" - ribonucleotide base ribocytosine.

Primer ID	Purpose	Sequence
**A. m_NR5A2**		
AcDx-5141-NR5A2-S1-FP	PCR	ATGTGCGGGTCGGCrGGGTC/3SpC3/
AcDx-5142-NR5A2-S1-RP	PCR	GGTGTCGTGGTCTACTCTCAACACCTCCCAArUCCTC/3SpC3/
AcDx-5143-NR5A2-S1-Up	LDR	TCATCGCCCTCAGATCTTCCAGGTCGGCGGGTTTGTGATCTCrG GAGT/3SpC3/
AcDx-5144-NR5A2-S1-Dn	LDR	/5Phos/GGAACGTTTTTTGTTATTTTTTTGCGCGAATTTGAAAGTG GAGGATAGATTGGAGGGCA
AcDx-5145-NR5A2-S1-RT-Pb	qPCR	/56-FAM/AATGATCTC/ZEN/GGAACGTTTTTTGTTATTTTTTTGCG/3IABkFQ/
AcDx-5146-NR5A2-S1-RT-FP	qPCR	TCATCGCCCTCAGATCTTCCA
AcDx-5147-NR5A2-S1-RT-RP	qPCR	TGCCCTCCAATCTATCCTCCA
**B. m_PRKCB**		
AcDx-5151-PRKCB-S1-FP	PCR	TTAAGCGTAGTTGGACGAGCrGGTAA/3SpC3/
AcDx-5152-PRKCB-S1-RP	PCR	GGTGTCGTGGTCCCCTACGCCGACTCrUAACA/3SpC3/
AcDx-5153-PRKCB-S1-Up	LDR	TTCGCCTACCGCAGTGAACACGAGCGGTAGTAGTTGAGCrGAG CA/3SpC3/
AcDx-5154-PRKCB-S1-Dn	LDR	/5Phos/GAGTGATAGTTTCGGTTTCGCGCGTCGGTTGAGACATGG GCTCGCA
AcDx-5155-PRKCB-S1-RT-Pb	qPCR	/56-FAM/ATGTTGAGC/ZEN/GAGTGATAGTTTCGGTTTCGC/3IABkFQ/
AcDx-5156-PRKCB-S1-RT-FP	qPCR	TTCGCCTACCGCAGTGAAC
AcDx-5157-PRKCB-S1-RT-RP	qPCR	TGCGAGCCCATGTCTCAAC
**C. m_ncr1**		
AcDx-5161-NCR1-S1-FP	PCR	TTTAAATTTAGGTTCGTTTAGTTATAGGCrGTTTT/3SpC3/
AcDx-5162-NCR1-S1-RP	PCR	GGTGTCGTGGACGCGCCCCGAAACrUAAAC/3SpC3/
AcDx-5163-NCR1-S1-Up	LDR	TGGATCGAGACGGAATGCAACGTTCGTTTAGTTATAGGCGTTT AGGTTTAATCrGGTCC/3SpC3/
AcDx-5164-NCR1-S1-Dn	LDR	/5Phos/GGTTTTTTTTATATAATTTTTTTTATAGTATGCGCGGGCG GGTTTCCCTGATTGATACCCGCA
AcDx-5165-NCR1-S1-RT-Pb	qPCR	/56-FAM/CATTTAATC/ZEN/GGTTTTTTTTATATAATTTTTTTTATAGT ATGCGCG/3IABkFQ/
AcDx-5166-NCR1-S1-RT-FP	qPCR	TGGATCGAGACGGAATGCAAC
AcDx-5167-NCR1-S1-RT-RP	qPCR	TGCGGGTATCAATCAGGGAAAC

**D. m_GRK7**		
AcDx-5221-GRK7-S1-FP	PCR	GGGTTTTAGGGTCGTATTTTTTCrGAGGC/3SpC3/
AcDx-5222-GRK7-S1-RP	PCR	GGTGTCGTGGTCTCCTTCCTTCCTCCGAArAAAAG/3SpC3/
AcDx-5223-GRK7-S1-Up	LDR	TTCGTGCGTCGTGTAGCAATCGAGGTTTGTAAAGGTTATTGTTT AAAAGCrGTAAG/3SpC3/
AcDx-5224-GRK7-S1-Dn	LDR	/5Phos/GTAGAGGAGTAGTTGGGAACGAGAATAAAGCGGTTGCC CATTTTCTGCACCCA
AcDx-5225-GRK7-S1-RT-Pb	qPCR	/56-FAM/AATAAAAGC/ZEN/GTAGAGGAGTAGTTGGGAACGA/3IABkFQ/
AcDx-5226-GRK7-S1-RT-FP	qPCR	TTCGTGCGTCGTGTAGCAA
AcDx-5227-GRK7-S1-RT-RP	qPCR	TGGGTGCAGAAAATGGGCAA

Supplement 3 (Table). Genomic information for the breast cancer-specific CpG sites interrogated in the multiplex PCR, LDR, qPCR assay described in this report.

CpG Marker ID	CpG ID	CpG_Coord(37)	Name	Relation_to_UCSC_CpG_Island	Enhancer
m_NR5A2	cg04097639	Chr1:_200009830_200009831	NR5A2	Island	
m_PRKCB	cg03306374	Chr16:_23847325_23847326	PRKCB	Island	
m_ncr1	cg16171526	Chr1:_155043744_155043745		Island	TRUE

Supplement 4 (Tables). A. Statistical summary of the β values (methylation level) for 3 CpG markers across major types of cancer. β value ranges from 0 (ummethylated) to 1 (completely methylated). PT = primary tumor; SN= solid normal. **B-D**. Statistical comparison (for each of the marker) between each subgroup.

A. Statistical Summaries for each marker in 7 major cancer types

			m_ncr1				m_NR5A2				m_PRKCB			
			PT		SN		PT		SN		PT		SN	
Cohort	# PT	#SN	Mean	St Dev	Mean	St Dev	Mean	St Dev	Mean	St Dev	Mean	St Dev	Mean	St Dev
BRCA	668	98	0.536	0.76	0.206	0.644	0.604	0.749	0.156	0.618	0.561	0.784	0.091	0.635
OV	79	6	0.048	0.511	0.05	0.521	0.163	0.671	0.224	0.634	0.065	0.63	0.062	0.552
UCEC	431	46	0.093	0.678	0.035	0.533	0.158	0.733	0.101	0.577	0.328	0.843	0.06	0.561
COADREAD	395	45	0.03	0.549	0.028	0.518	0.163	0.746	0.046	0.514	0.362	0.826	0.023	0.509
LUAD	458	32	0.071	0.571	0.066	0.522	0.454	0.738	0.106	0.526	0.446	0.757	0.054	0.53
LUSC	372	43	0.054	0.603	0.031	0.511	0.436	0.78	0.055	0.52	0.112	0.709	0.019	0.507
PAAD	184	10	0.029	0.519	0.03	0.51	0.139	0.699	0.026	0.51	0.498	0.719	0.076	0.659

B. m_ncr1 methylation level statistical comparison (t test) between each subgroup

Two subgroups compared		Difference	Std Err Dif	Lower CL	Upper CL	p-Value
**BRCA_PT**	**BRCA_SN**	0.3299201	0.016722	0.297132	0.3627087	<.0001
**BRCA_PT**	**OV_PT**	0.4884582	0.0183923	0.452395	0.5245216	<.0001
**BRCA_PT**	**OV_SN**	0.486162	0.0633932	0.361861	0.610463	<.0001
**BRCA_PT**	**UCEC_PT**	0.4434017	0.009551	0.424674	0.4621293	<.0001
**BRCA_PT**	**UCEC_SN**	0.5008598	0.0235645	0.454655	0.547065	<.0001
**BRCA_PT**	**COADREAD_PT**	0.5060908	0.009812	0.486852	0.5253301	<.0001
**BRCA_PT**	**COADREAD_SN**	0.5077486	0.0238082	0.461066	0.5544317	<.0001
**BRCA_PT**	**LUAD_PT**	0.4650661	0.0093783	0.446677	0.4834551	<.0001
**BRCA_PT**	**LUAD_SN**	0.4699401	0.0279745	0.415088	0.5247924	<.0001
**BRCA_PT**	**LUSC_PT**	0.4817958	0.0100008	0.462186	0.5014053	<.0001
**BRCA_PT**	**LUSC_SN**	0.504662	0.0243214	0.456973	0.5523513	<.0001
**BRCA_PT**	**PAAD_PT**	0.5071473	0.0128706	0.481911	0.5323839	<.0001
**BRCA_PT**	**PAAD_SN**	0.505772	0.0492496	0.409204	0.6023404	<.0001

C. m_NR5A2 methylation level statistical comparison (t test) between each subgroup

Two subgroups compared		Difference	Std Err Dif	Lower CL	Upper CL	p-Value
**BRCA_PT**	**BRCA_SN**	0.4489162	0.025206	0.399492	0.49834	<.0001
**BRCA_PT**	**OV_PT**	0.441232	0.027724	0.386872	0.495592	<.0001
**BRCA_PT**	**OV_SN**	0.3805213	0.095556	0.193156	0.567887	<.0001
**BRCA_PT**	**UCEC_PT**	0.4467789	0.014397	0.41855	0.475008	<.0001
**BRCA_PT**	**UCEC_SN**	0.5035147	0.03552	0.433867	0.573162	<.0001

**BRCA_PT**	**COADREAD_PT**	0.4413061	0.01479	0.412306	0.470306	<.0001
**BRCA_PT**	**COADREAD_SN**	0.5581535	0.035887	0.487786	0.628521	<.0001
**BRCA_PT**	**LUAD_PT**	0.15064	0.014136	0.122921	0.178359	<.0001
**BRCA_PT**	**LUAD_SN**	0.4983681	0.042167	0.415687	0.58105	<.0001
**BRCA_PT**	**LUSC_PT**	0.1679871	0.015075	0.138429	0.197546	<.0001
**BRCA_PT**	**LUSC_SN**	0.5494108	0.036661	0.477526	0.621295	<.0001
**BRCA_PT**	**PAAD_PT**	0.4650147	0.019401	0.426974	0.503055	<.0001
**BRCA_PT**	**PAAD_SN**	0.5789413	0.074237	0.433379	0.724504	<.0001

C. m_PRKCB methylation level statistical comparison (t test) between each subgroup

**Two subgroups compared**		**Difference**	**Std Err Dif**	**Lower CL**	**Upper CL**	**p-Value**
**BRCA_PT**	**BRCA_SN**	0.4694573	0.029	0.412594	0.52632	<.0001
**BRCA_PT**	**OV_PT**	0.4955842	0.031896	0.433042	0.558127	<.0001
**BRCA_PT**	**OV_SN**	0.4983015	0.109939	0.282735	0.713869	<.0001
**BRCA_PT**	**UCEC_PT**	0.2328358	0.016564	0.200358	0.265314	<.0001
**BRCA_PT**	**UCEC_SN**	0.5007631	0.040866	0.420633	0.580894	<.0001
**BRCA_PT**	**COADREAD_PT**	0.1980836	0.017016	0.164718	0.231449	<.0001
**BRCA_PT**	**COADREAD_SN**	0.5377727	0.041289	0.456813	0.618732	<.0001
**BRCA_PT**	**LUAD_PT**	0.1146623	0.016264	0.082772	0.146553	<.0001
**BRCA_PT**	**LUAD_SN**	0.5067911	0.048514	0.411665	0.601918	<.0001
**BRCA_PT**	**LUSC_PT**	0.4484663	0.017344	0.414459	0.482474	<.0001
**BRCA_PT**	**LUSC_SN**	0.5410907	0.042179	0.458386	0.623795	<.0001
**BRCA_PT**	**PAAD_PT**	0.0624903	0.022321	0.018724	0.106257	0.0051
**BRCA_PT**	**PAAD_SN**	0.4841949	0.08541	0.316723	0.651667	<.0001

Supplement 5 (Figure and Tables). Relationship between CpG methylation and Primary Tumor Stage.

Part A. Comparative level of methylation (β value) at m_ncr1, m_PRKCB, and m_NR5A2 in normal breast tissues, and each of the 3 primary tumor stages of breast cancer. The β values range from -0.5 (0%methyaltion) to +0.5 (100% methylation).

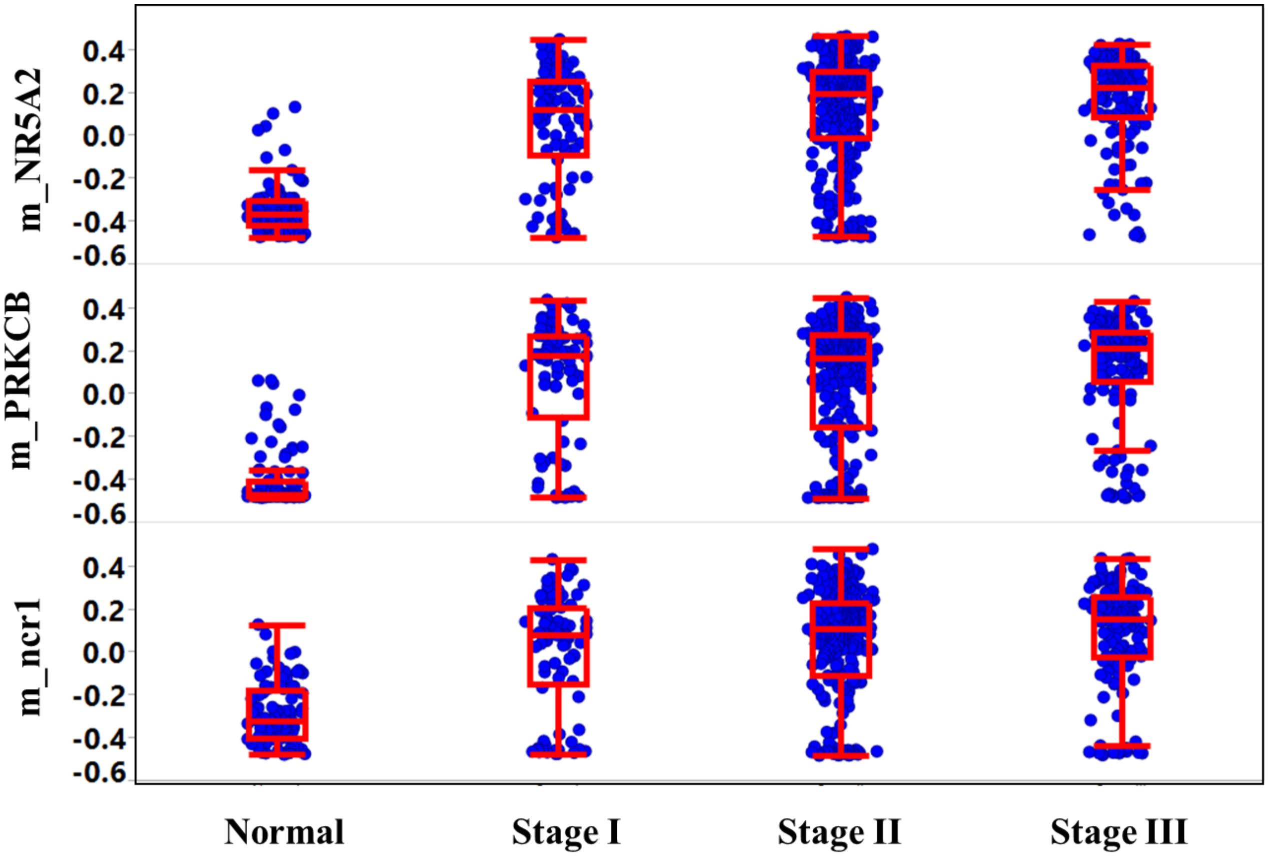

Part B. Comparison of means for m_ncr1

Level	Number	Mean	Std Dev	Std Err Mean	Lower 95%	Upper 95%
Normal	96	-0.29493	0.145152	0.01481	-0.3243	-0.2655
Stage I	89	-0.00072	0.274439	0.02909	-0.0585	0.0571
Stage II	309	0.03442	0.265761	0.01512	0.0047	0.0642
Stage III	136	0.09018	0.237303	0.02035	0.0499	0.1304

Ordered Differences Report

Level	- Level	Difference	Std Err Dif	Lower CL	Upper CL	p-Value
Stage III	Normal	0.3851105	0.0328367	0.320627	0.4495940	<.0001*
Stage II	Normal	0.3293511	0.0287828	0.272829	0.3858737	<.0001*
Stage I	Normal	0.2942176	0.0362473	0.223037	0.3653987	<.0001*
Stage III	Stage I	0.0908929	0.0335852	0.024940	0.1568462	0.0070*
Stage III	Stage II	0.0557594	0.0253485	0.005981	0.1055378	0.0282*
Stage II	Stage I	0.0351335	0.0296339	-0.023060	0.0933273	0.2362

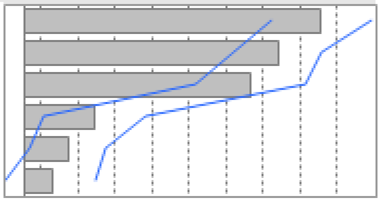

Part C. Comparison of means for m_NR5A2

Level	Number	Mean	Std Dev	Std Err Mean	Lower 95%	Upper 95%
Normal	96	-0.34368	0.119312	0.01218	-0.3679	-0.3195
Stage I	89	0.05237	0.264735	0.02806	-0.0034	0.1081
Stage II	309	0.11018	0.251557	0.01431	0.0820	0.1383
Stage III	136	0.16222	0.218533	0.01874	0.1252	0.1993

Ordered Differences Report

Level	- Level	Difference	Std Err Dif	Lower CL	Upper CL	p-Value
Stage III	Normal	0.5059030	0.0308175	0.4453847	0.5664213	<.0001*
Stage II	Normal	0.4538647	0.0270129	0.4008177	0.5069116	<.0001*
Stage I	Normal	0.3960532	0.0340184	0.3292492	0.4628573	<.0001*
Stage III	Stage I	0.1098498	0.0315200	0.0479521	0.1717474	0.0005*
Stage II	Stage I	0.0578114	0.0278116	0.0031961	0.1124268	0.0381*
Stage III	Stage II	0.0520383	0.0237898	0.0053209	0.0987558	0.0291*

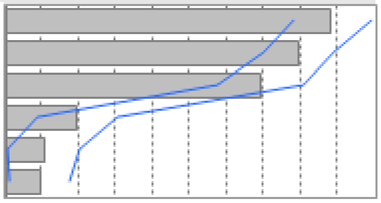

Part D. Comparison of means for m_PRKCB

Level	Number	Mean	Std Dev	Std Err Mean	Lower 95%	Upper 95%
Normal	96	-0.40747	0.136051	0.01389	-0.4350	-0.3799
Stage I	89	0.06242	0.284425	0.03015	0.0025	0.1223
Stage II	309	0.04843	0.298065	0.01696	0.0151	0.0818
Stage III	136	0.11100	0.253564	0.02174	0.0680	0.1540

Ordered Differences Report

Level	- Level	Difference	Std Err Dif	Lower CL	Upper CL	p-Value
Stage III	Normal	0.5184703	0.0357088	0.448347	0.5885938	<.0001*
Stage I	Normal	0.4698953	0.0394177	0.392488	0.5473023	<.0001*
Stage II	Normal	0.4559008	0.0313003	0.394434	0.5173672	<.0001*
Stage III	Stage II	0.0625695	0.0275656	0.008437	0.1167017	0.0236*
Stage III	Stage I	0.0485750	0.0365227	-0.023147	0.1202968	0.1840
Stage I	Stage II	0.0139945	0.0322258	-0.049289	0.0772782	0.6642

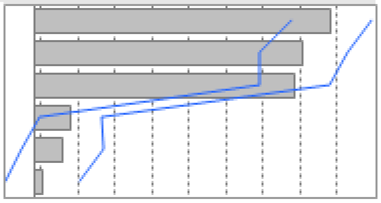

Supplement 6 (Figures and Tables). Relationship between CpG methylation and molecular subtypes of breast cancer.

A. Comparative level of methylation (β value) at m_ncr1, m_PRKCB, and m_NR5A2 in normal breast tissues, and each of the 4 molecular subtypes of breast cancer. The β values range from -0.5 (0%methyaltion) to +0.5 (100% methylation).

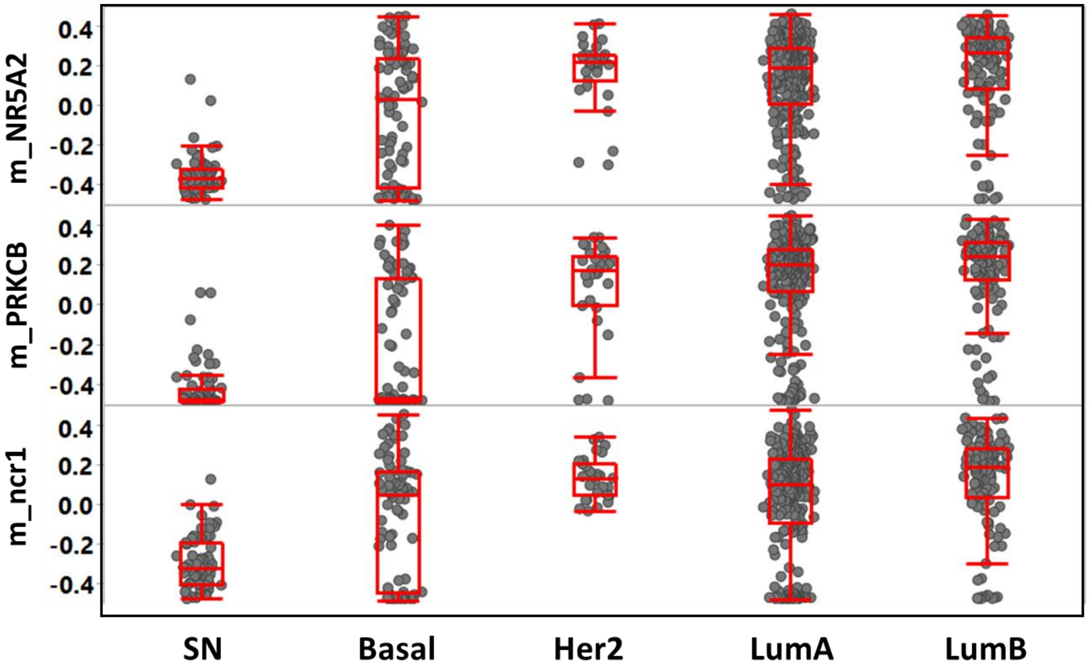

B. Comparison of means for m_ncr1

Level	Number	Mean	Std Dev	Std Err Mean	Lower 95%	Upper 95%
Basal	84	-0.05970	0.303004	0.03306	-0.1255	0.0061
Her2	31	0.13038	0.103001	0.01850	0.0926	0.1682
LumA	275	0.03160	0.263412	0.01588	0.00033	0.0629
LumB	125	0.12785	0.235750	0.02109	0.0861	0.1696
Solid Normal	69	-0.29641	0.134018	0.01613	-0.3286	-0.2642

Ordered Differences Report

Level	- Level	Difference	Std Err Dif	Lower CL	Upper CL	p-Value
Her2	Solid Normal	0.4267951	0.0532087	0.322290	0.5313007	<.0001*
LumB	Solid Normal	0.4242633	0.0369070	0.351775	0.4967513	<.0001*
LumA	Solid Normal	0.3280156	0.0331342	0.262938	0.3930934	<.0001*
Basal	Solid Normal	0.2367145	0.0399825	0.158186	0.3152428	<.0001*
Her2	Basal	0.1900806	0.0517150	0.088509	0.2916525	0.0003*
LumB	Basal	0.1875488	0.0347189	0.119358	0.2557392	<.0001*
Her2	LumA	0.0987796	0.0466231	0.007208	0.1903506	0.0345*
LumB	LumA	0.0962477	0.0265458	0.044110	0.1483856	0.0003*
LumA	Basal	0.0913011	0.0306782	0.031047	0.1515551	0.0030*
Her2	LumB	0.0025318	0.0493758	-0.094446	0.0995094	0.9591

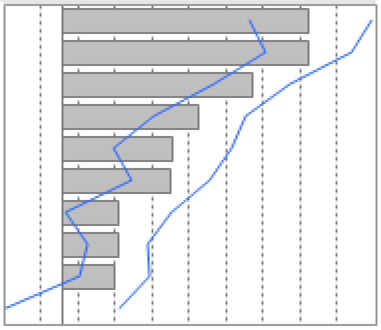

C. Comparison of means for m_NR5A2

Level	Number	Mean	Std Dev	Std Err Mean	Lower 95%	Upper 95%
Basal	84	-0.03335	0.318156	0.03471	-0.1024	0.0357
Her2	31	0.17084	0.175765	0.03157	0.1064	0.2353
LumA	275	0.12894	0.222703	0.01343	0.1025	0.1554
LumB	125	0.18730	0.219275	0.01961	0.1485	0.2261
Solid Normal	69	-0.35233	0.100427	0.01209	-0.3765	-0.3282

Ordered Differences Report

Level	- Level	Difference	Std Err Dif	Lower CL	Upper CL	p-Value
LumB	Solid Normal	0.5396298	0.0338903	0.473067	0.6061928	<.0001*
Her2	Solid Normal	0.5231645	0.0488595	0.427201	0.6191280	<.0001*
LumA	Solid Normal	0.4812650	0.0304259	0.421506	0.5410235	<.0001*
Basal	Solid Normal	0.3189790	0.0367144	0.246869	0.3910886	<.0001*
LumB	Basal	0.2206508	0.0318811	0.158034	0.2832675	<.0001*
Her2	Basal	0.2041855	0.0474879	0.110916	0.2974550	<.0001*
LumA	Basal	0.1622860	0.0281706	0.106957	0.2176150	<.0001*
LumB	LumA	0.0583648	0.0243760	0.010489	0.1062411	0.0170*
Her2	LumA	0.0418995	0.0428123	-0.042187	0.1259857	0.3281
LumB	Her2	0.0164653	0.0453400	-0.072586	0.1055161	0.7166

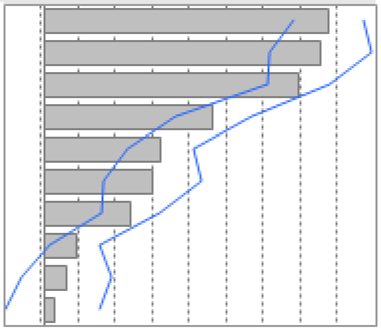

D. Comparison of means for m_PRKCB

Level	Number	Mean	Std Dev	Std Err Mean	Lower 95%	Upper 95%
Basal	84	-0.22185	0.319651	0.03488	-0.2912	-0.1525
Her2	31	0.08782	0.241130	0.04331	-0.0006	0.1763
LumA	275	0.12627	0.234603	0.01415	0.0984	0.1541
LumB	125	0.17095	0.216626	0.01938	0.1326	0.2093
Solid Normal	69	-0.42430	0.114567	0.01379	-0.4518	-0.3968

Ordered Differences Report

Level	- Level	Difference	Std Err Dif	Lower CL	Upper CL	p-Value
LumB	Solid Normal	0.5952450	0.0352673	0.525977	0.6645125	<.0001*
LumA	Solid Normal	0.5505655	0.0316621	0.488379	0.6127520	<.0001*
Her2	Solid Normal	0.5121179	0.0508447	0.412255	0.6119805	<.0001*
LumB	Basal	0.3927928	0.0331765	0.327632	0.4579537	<.0001*
LumA	Basal	0.3481133	0.0293152	0.290536	0.4056904	<.0001*
Her2	Basal	0.3096658	0.0494174	0.212607	0.4067250	<.0001*
Basal	Solid Normal	0.2024521	0.0382061	0.127413	0.2774916	<.0001*
LumB	Her2	0.0831270	0.0471822	-0.009542	0.1757961	0.0786
LumB	LumA	0.0446795	0.0253665	-0.005142	0.0945010	0.0787
LumA	Her2	0.0384476	0.0445518	-0.049055	0.1259503	0.3885

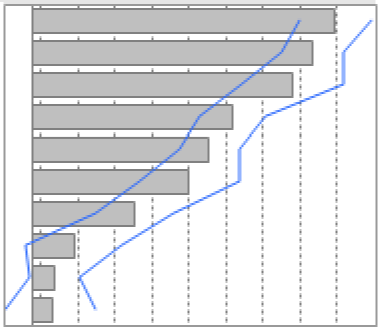

Supplement 7 (Figures and Tables). Relationship between CpG methylation and patient age.

A. m_NR5A2 v. Age of Diagnosis

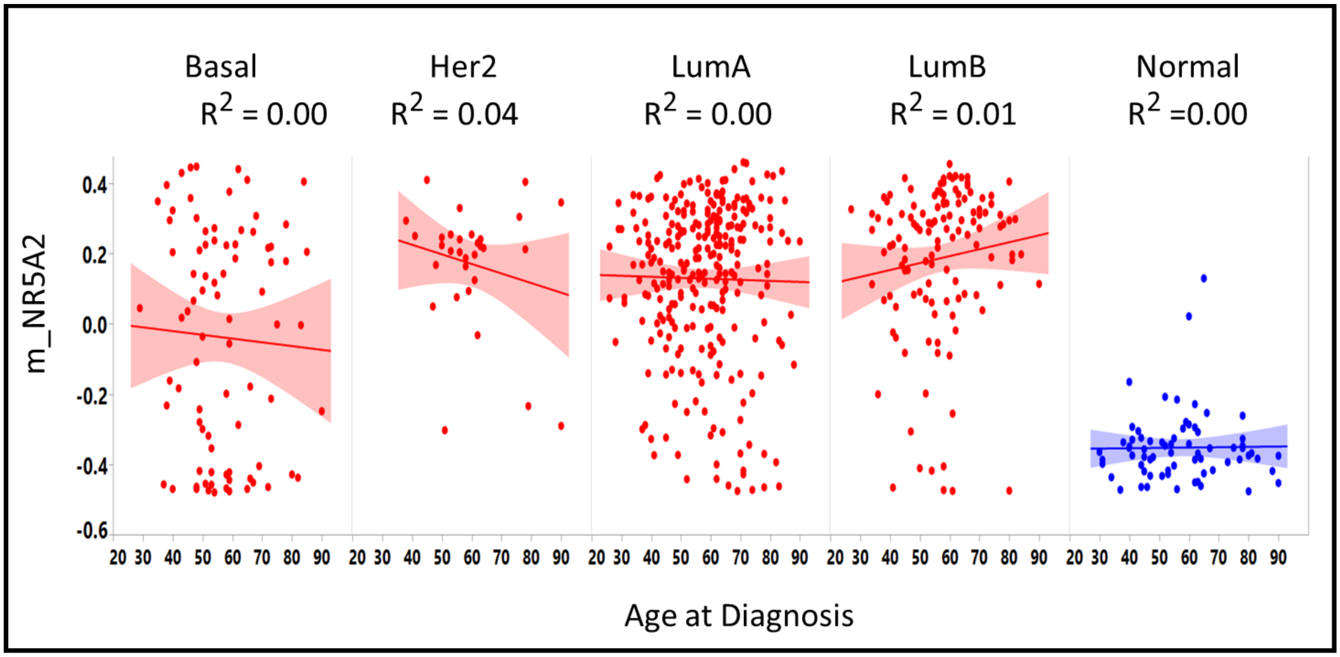

Bivariate analyses for m_NR5A2 v. Age of Diagnosis for every molecular subtype of breast cancer

1. Basal

Linear Fit
m_NR5A2 = 0.0224009 - 0.0010693*Age

Summary of Fit

RSquare	0.001961
RSquare Adj	-0.01036
Root Mean Square Error	0.318466
Mean of Response	-0.03829
Observations (or Sum Wgts)	83

Analysis of Variance

Source	DF	Sum of Squares	Mean Square	F Ratio
Model	1	0.0161446	0.016145	0.1592
Error	81	8.2150447	0.101420	**Prob > F**
C. Total	82	8.2311893		0.6910

Parameter Estimates

Term	Estimate	Std Error	t Ratio	Prob>|t|
Intercept	0.0224009	0.156087	0.14	0.8862
Age	-0.001069	0.00268	-0.40	0.6910

2. Her2

Linear Fit
m_NR5A2 = 0.3347712 - 0.0027308*Age

Summary of Fit

RSquare	0.039853
RSquare Adj	0.006744
Root Mean Square Error	0.175171
Mean of Response	0.170835
Observations (or Sum Wgts)	31

Analysis of Variance

Source	DF	Sum of Squares	Mean Square	F Ratio
Model	1	0.03693545	0.036935	1.2037
Error	29	0.88986060	0.030685	**Prob > F**
C. Total	30	0.92679605		0.2816

Parameter Estimates

Term	Estimate	Std Error	t Ratio	Prob>|t|
Intercept	0.3347712	0.152698	2.19	0.0365*
Age	-0.002731	0.002489	-1.10	0.2816

3. LumA

Linear Fit
m_NR5A2 = 0.1458252 - 0.0002915*Age

Summary of Fit

RSquare	0.000311
RSquare Adj	-0.00335
Root Mean Square Error	0.223075
Mean of Response	0.128936
Observations (or Sum Wgts)	275

Analysis of Variance

Source	DF	Sum of Squares	Mean Square	F Ratio
Model	1	0.004233	0.004233	0.0851
Error	273	13.585201	0.049763	**Prob > F**
C. Total	274	13.589433		0.7708

Parameter Estimates

Term	Estimate	Std Error	t Ratio	Prob>|t|
Intercept	0.1458252	0.059453	2.45	0.0148*
Age	-0.000291	0.000999	-0.29	0.7708

4. LumB

Linear Fit
m_NR5A2 = 0.0744462 + 0.001978*Age

Summary of Fit

RSquare	0.013055
RSquare Adj	0.005031
Root Mean Square Error	0.218723
Mean of Response	0.187301
Observations (or Sum Wgts)	125

Analysis of Variance

Source	DF	Sum of Squares	Mean Square	F Ratio
Model	1	0.0778344	0.077834	1.6270
Error	123	5.8842656	0.047840	**Prob > F**
C. Total	124	5.9621000		0.2045

Parameter Estimates

Term	Estimate	Std Error	t Ratio	Prob>|t|
Intercept	0.0744462	0.090613	0.82	0.4129
Age	0.001978	0.001551	1.28	0.2045

5. Normal

Linear Fit
m_NR5A2 = -0.357937 + 0.0000979*Age

Summary of Fit

RSquare	0.000221
RSquare Adj	-0.0147
Root Mean Square Error	0.101163
Mean of Response	-0.35233
Observations (or Sum Wgts)	69

Analysis of Variance

Source	DF	Sum of Squares	Mean Square	F Ratio
Model	1	0.00015122	0.000151	0.0148
Error	67	0.68567118	0.010234	**Prob > F**
C. Total	68	0.68582240		0.9036

Parameter Estimates

Term	Estimate	Std Error	t Ratio	Prob>|t|
Intercept	-0.357937	0.047717	-7.50	<.0001*
Age	0.0000979	0.000805	0.12	0.9036

B. m_ncr1

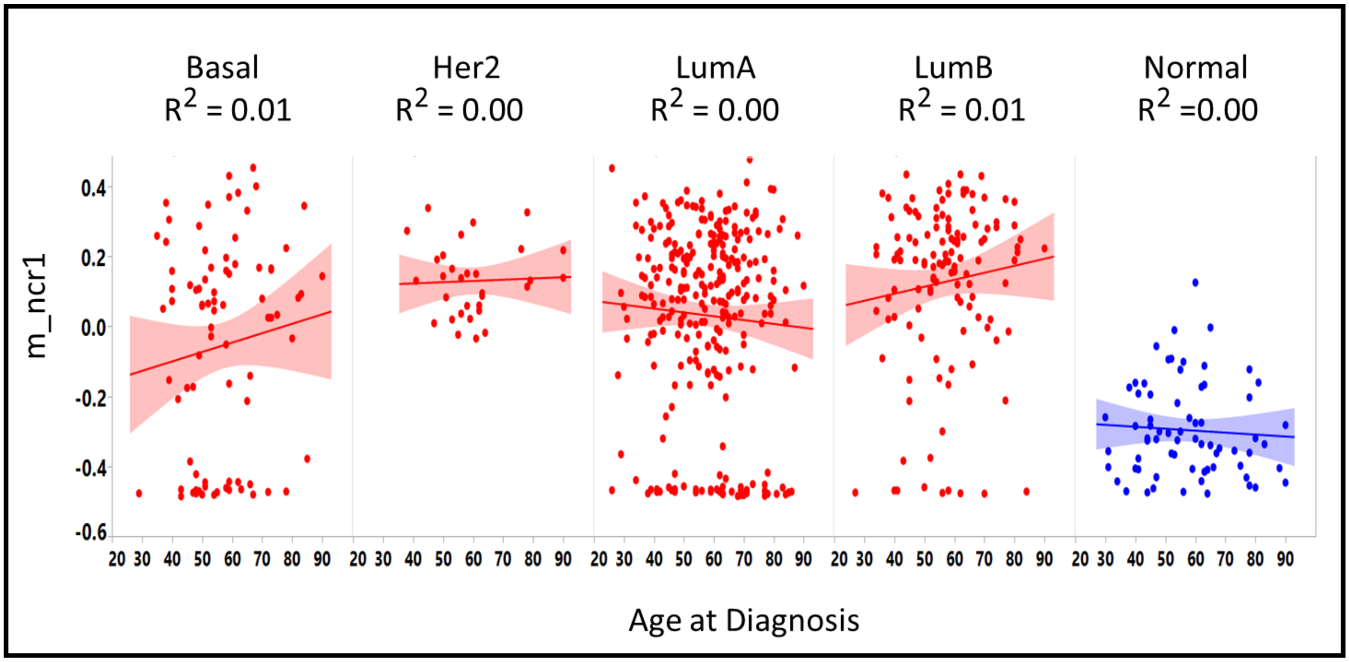

Bivariate analyses for m_ncr1 v. age of diagnosis for every molecular subtype of breast cancer

1. Basal

Linear Fit
m_ncr1 = -0.20773 + 0.0026946*Age

Summary of Fit

RSquare	0.013757
RSquare Adj	0.001581
Root Mean Square Error	0.301223
Mean of Response	-0.05479
Observations (or Sum Wgts)	83

Analysis of Variance

Source	DF	Sum of Squares	Mean Square	F Ratio
Model	1	0.1025176	0.102518	1.1299
Error	81	7.3495446	0.090735	**Prob > F**
C. Total	82	7.4520623		0.2910

Parameter Estimates

Term	Estimate	Std Error	t Ratio	Prob>|t|
Intercept	-0.20773	0.147636	-1.41	0.1632
Age	0.0026946	0.002535	1.06	0.2910

2. Her2

Linear Fit
m_ncr1 = 0.1090932 + 0.0003546*Age

Summary of Fit

RSquare	0.001957
RSquare Adj	-0.03246
Root Mean Square Error	0.104659
Mean of Response	0.130381
Observations (or Sum Wgts)	31

Analysis of Variance

Source	DF	Sum of Squares	Mean Square	F Ratio
Model	1	0.00062279	0.000623	0.0569
Error	29	0.31765176	0.010954	**Prob > F**
C. Total	30	0.31827455		0.8132

Parameter Estimates

Term	Estimate	Std Error	t Ratio	Prob>|t|
Intercept	0.1090932	0.091232	1.20	0.2415
Age	0.0003546	0.001487	0.24	0.8132

3. LumA

Linear Fit
m_ncr1 = 0.0951138 - 0.0010961*Age

Summary of Fit

RSquare	0.003148
RSquare Adj	-0.0005
Root Mean Square Error	0.263479
Mean of Response	0.031601
Observations (or Sum Wgts)	275

Analysis of Variance

Source	DF	Sum of Squares	Mean Square	F Ratio
Model	1	0.059856	0.059856	0.8622
Error	273	18.951919	0.069421	**Prob > F**
C. Total	274	19.011774		0.3539

Parameter Estimates

Term	Estimate	Std Error	t Ratio	Prob>|t|
Intercept	0.0951138	0.070221	1.35	0.1767
Age	-0.001096	0.00118	-0.93	0.3539

4. LumB

Linear Fit
m_ncr1 = 0.0136965 + 0.0020007*Age

Summary of Fit

RSquare	0.011555
RSquare Adj	0.003519
Root Mean Square Error	0.235335
Mean of Response	0.127849
Observations (or Sum Wgts)	125

Analysis of Variance

Source	DF	Sum of Squares	Mean Square	F Ratio
Model	1	0.0796346	0.079635	1.4379
Error	123	6.8120618	0.055383	**Prob > F**
C. Total	124	6.8916964		0.2328

Parameter Estimates

Term	Estimate	Std Error	t Ratio	Prob>|t|
Intercept	0.0136965	0.097496	0.14	0.8885
Age	0.0020007	0.001668	1.20	0.2328

5. Normal

Linear Fit
m_ncr1 = -0.264467 - 0.0005576*Age

Summary of Fit

RSquare	0.004018
RSquare Adj	-0.01085
Root Mean Square Error	0.134742
Mean of Response	-0.29641
Observations (or Sum Wgts)	69

Analysis of Variance

Source	DF	Sum of Squares	Mean Square	F Ratio
Model	1	0.0049071	0.004907	0.2703
Error	67	1.2164201	0.018156	**Prob > F**
C. Total	68	1.2213271		0.6049

Parameter Estimates

Term	Estimate	Std Error	t Ratio	Prob>|t|
Intercept	-0.264467	0.063555	-4.16	<.0001*
Age	-0.000558	0.001073	-0.52	0.6049

C. m_PRKCB

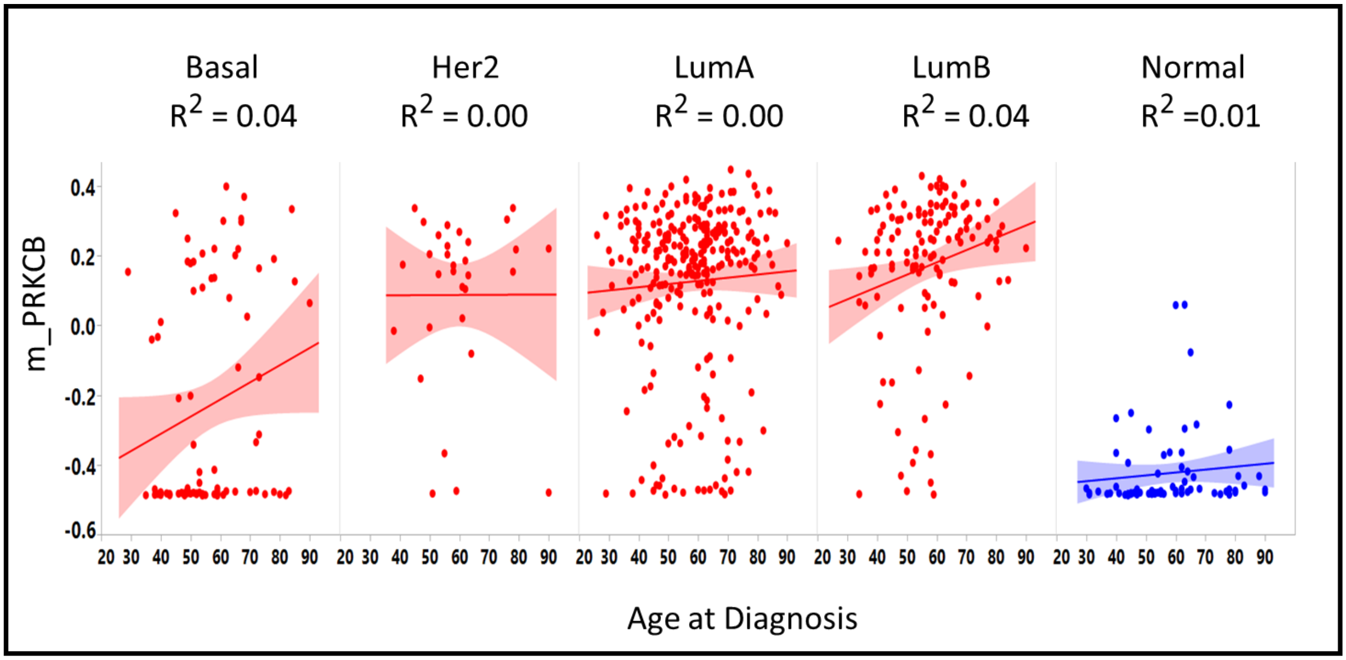

Bivariate analyses for m_PRKCB v. age of diagnosis for every molecular subtype of breast cancer

1. Basal

Linear Fit
m_PRKCB = -0.508481 + 0.0049357*Age

Summary of Fit

RSquare	0.042012
RSquare Adj	0.030185
Root Mean Square Error	0.311175
Mean of Response	-0.22833
Observations (or Sum Wgts)	83

Analysis of Variance

Source	DF	Sum of Squares	Mean Square	F Ratio
Model	1	0.3439599	0.343960	3.5522
Error	81	7.8432045	0.096830	**Prob > F**
C. Total	82	8.1871644		0.0631

Parameter Estimates

Term	Estimate	Std Error	t Ratio	Prob>|t|
Intercept	-0.508481	0.152513	-3.33	0.0013*
Age	0.0049357	0.002619	1.88	0.0631

2. HER2

Linear Fit
m_PRKCB = 0.0852113 + 4.3445e-5*Age

Summary of Fit

RSquare	5.359e-6
RSquare Adj	-0.03448
Root Mean Square Error	0.245251
Mean of Response	0.087819
Observations (or Sum Wgts)	31

Analysis of Variance

Source	DF	Sum of Squares	Mean Square	F Ratio
Model	1	0.0000093	9.348e-6	0.0002
Error	29	1.7442988	0.060148	**Prob > F**
C. Total	30	1.7443081		0.9901

Parameter Estimates

Term	Estimate	Std Error	t Ratio	Prob>|t|
Intercept	0.0852113	0.213788	0.40	0.6931
Age	4.3445e-5	0.003485	0.01	0.9901

3. LumA

Linear Fit
m_PRKCB = 0.073532 + 0.0009101*Age

Summary of Fit

RSquare	0.002736
RSquare Adj	-0.00092
Root Mean Square Error	0.234711
Mean of Response	0.126267
Observations (or Sum Wgts)	275

Analysis of Variance

Source	DF	Sum of Squares	Mean Square	F Ratio
Model	1	0.041265	0.041265	0.7491
Error	273	15.039324	0.055089	**Prob > F**
C. Total	274	15.080589		0.3875

Parameter Estimates

Term	Estimate	Std Error	t Ratio	Prob>|t|
Intercept	0.073532	0.062554	1.18	0.2408
Age	0.0009101	0.001052	0.87	0.3875

4. LumB

Linear Fit
m_PRKCB = -0.031157 + 0.0035422*Age

Summary of Fit

RSquare	0.042898
RSquare Adj	0.035117
Root Mean Square Error	0.212789
Mean of Response	0.170946
Observations (or Sum Wgts)	125

Analysis of Variance

Source	DF	Sum of Squares	Mean Square	F Ratio
Model	1	0.2496212	0.249621	5.5130
Error	123	5.5693221	0.045279	**Prob > F**
C. Total	124	5.8189433		0.0205*

Parameter Estimates

Term	Estimate	Std Error	t Ratio	Prob>|t|
Intercept	-0.031157	0.088155	-0.35	0.7244
Age	0.0035422	0.001509	2.35	0.0205*

<u>5. Normal</u>

Linear Fit
m_PRKCB = -0.471957 + 0.0008319*Age

Summary of Fit

RSquare	0.012235
RSquare Adj	-0.00251
Root Mean Square Error	0.11471
Mean of Response	-0.4243
Observations (or Sum Wgts)	69

Analysis of Variance

Source	DF	Sum of Squares	Mean Square	F Ratio
Model	1	0.01092034	0.010920	0.8299
Error	67	0.88161585	0.013158	**Prob > F**
C. Total	68	0.89253619		0.3656

Parameter Estimates

Term	Estimate	Std Error	t Ratio	Prob>|t|
Intercept	-0.471957	0.054107	-8.72	<.0001*
Age	0.0008319	0.000913	0.91	0.3656

Supplement 8 (Figure and Tables). Relationship between CpG methylation and patient race

Part A. Comparative level of methylation (β value) at m_ncr1, m_PRKCB, and m_NR5A2 in normal both normal and primary tumor breast tissues, among 3 races. The β values range from -0.5 (0%methyaltion) to +0.5 (100% methylation).

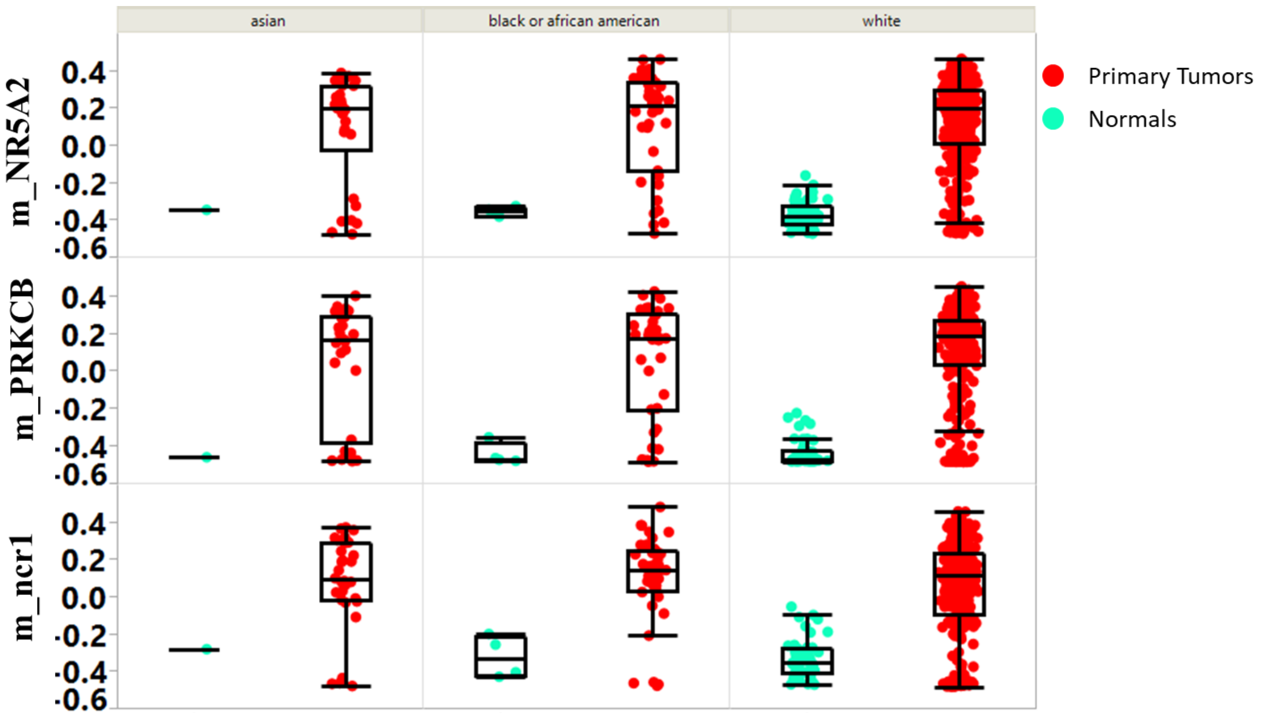

Part B. Comparison of means for m_ncr1

Ordered Differences Report

Level	- Level	Difference	Std Err Dif	Lower CL	Upper CL	p-Value
black or african american	white	0.0586910	0.0441624	-0.028100	0.1454817	0.1845
asian	white	0.0332169	0.0498131	-0.064679	0.1311126	0.5052
black or african american	asian	0.0254741	0.0638057	-0.099921	0.1508691	0.6899

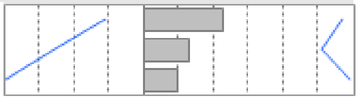

Part C. Comparison of means for m_NR5A2

Ordered Differences Report

Level	- Level	Difference	Std Err Dif	Lower CL	Upper CL	p-Value
white	asian	0.0330464	0.0469324	-0.059188	0.1252810	0.4817
black or african american	asian	0.0258269	0.0601160	-0.092317	0.1439705	0.6677
white	black or african american	0.0072195	0.0416086	-0.074552	0.0889912	0.8623

Part D. Comparison of means for m_PRKCB

Ordered Differences Report

Level	- Level	Difference	Std Err Dif	Lower CL	Upper CL	p-Value
white	asian	0.0490740	0.0518659	-0.052856	0.1510042	0.3446
white	black or african american	0.0396979	0.0459824	-0.050670	0.1300654	0.3884
black or african american	asian	0.0093762	0.0664353	-0.121187	0.1399389	0.8878

Supplement 9 (Tables with Figures). Relationship between CpG methylation and patient ethnicity .

Part A. Comparison of means for m_ncr1

Level	Number	Mean	Std Dev	Std Err Mean	Lower 95%	Upper 95%
hispanic or latino	25	-0.07489	0.311554	0.06231	-0.2035	0.05372
not hispanic or latino	431	0.05029	0.258432	0.01245	0.0258	0.07476

t Test

not hispanic or latino-hispanic or latino

Assuming unequal variances

Difference	0.12518	t Ratio	1.970058
Std Err Dif	0.06354	DF	25.95164
Upper CL Dif	0.25581	Prob > |t|	0.0596
Lower CL Dif	-0.00544	Prob > t	0.0298*
Confidence	0.95	Prob < t	0.9702

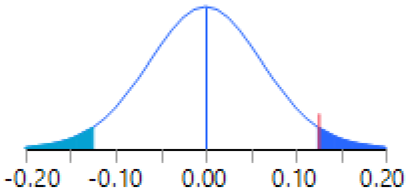

Part B. Comparison of means for m_NR5A2

Level	Number	Mean	Std Dev	Std Err Mean	Lower 95%	Upper 95%
hispanic or latino	25	0.127324	0.222628	0.04453	0.03543	0.21922
not hispanic or latino	431	0.119109	0.247983	0.01194	0.09563	0.14259

t Test

not hispanic or latino-hispanic or latino

Assuming unequal variances

Difference	-0.00821	t Ratio	-0.1782
Std Err Dif	0.04610	DF	27.57088
Upper CL Dif	0.08628	Prob > |t|	0.8599
Lower CL Dif	-0.10271	Prob > t	0.5701
Confidence	0.95	Prob < t	0.4299

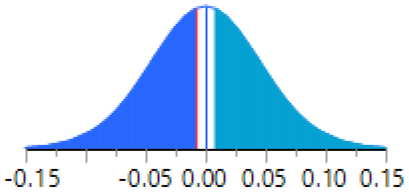

Part C. Comparison of means for m_PRKCB

Level	Number	Mean	Std Dev	Std Err Mean	Lower 95%	Upper 95%
hispanic or latino	25	0.037644	0.321016	0.06420	-0.0949	0.17015

Level	Number	Mean	Std Dev	Std Err Mean	Lower 95%	Upper 95%
not hispanic or latino	431	0.086284	0.270516	0.01303	0.0607	0.11189

t Test

not hispanic or latino-hispanic or latino
Assuming unequal variances

Difference	0.04864	t Ratio	0.742453
Std Err Dif	0.06551	DF	26.01539
Upper CL Dif	0.18330	Prob > |t|	0.4645
Lower CL Dif	-0.08602	Prob > t	0.2322
Confidence	0.95	Prob < t	0.7678

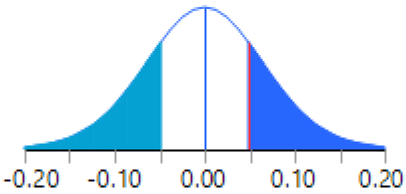

Supplement 10 (Figure). Relationship between CpG methylation and gene expression

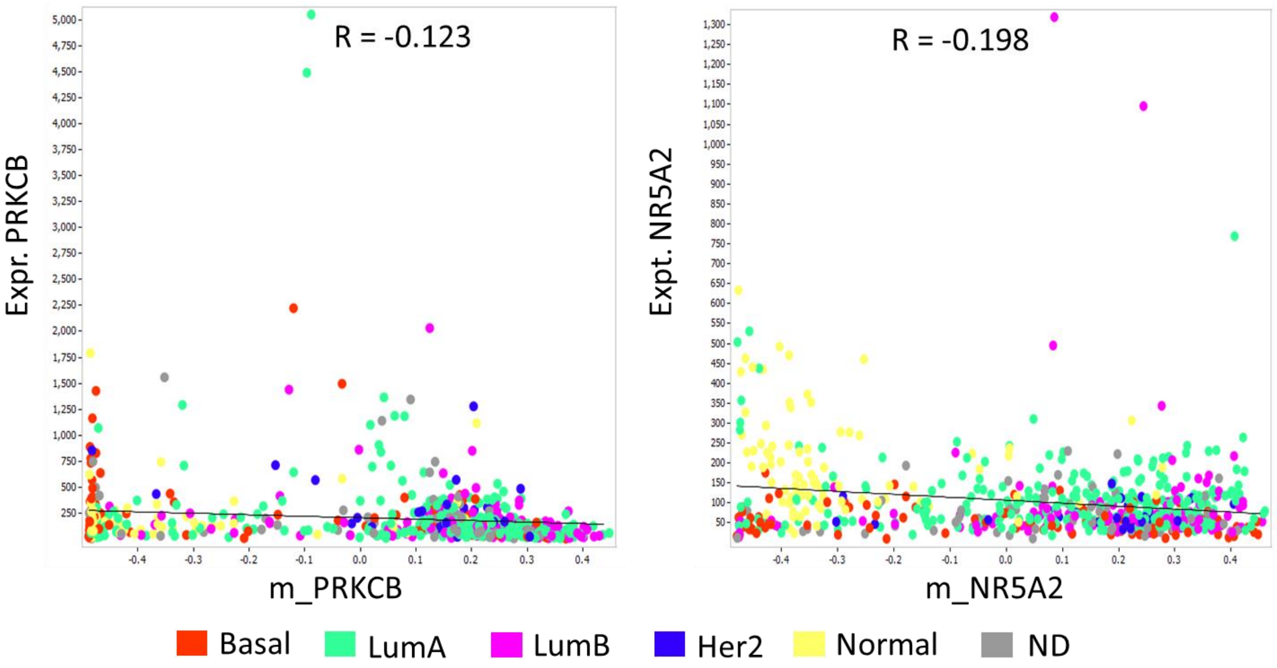

Linear Fit
Exp_NR5A2 = 6.3189526 - 0.7178041*m_NR5A2
Summary of Fit

RSquare	0.03604
RSquare Adj	0.034624
Root Mean Square Error	1.037971
Mean of Response	6.280387
Observations (or Sum Wgts)	683

Analysis of Variance

Source	DF	Sum of Squares	Mean Square	F Ratio
Model	1	27.43110	27.4311	25.4608
Error	681	733.69864	1.0774	**Prob > F**
C. Total	682	761.12973		<.0001*

Parameter Estimates

Term	Estimate	Std Error	t Ratio	Prob>|t|
Intercept	6.3189526	0.040446	156.23	<.0001*
m_NR5A2	-0.717804	0.142256	-5.05	<.0001*

Linear Fit
Exp_PRKCB = 6.8118407 - 0.9594483*m_PRKCB
Summary of Fit

RSquare	0.039227
RSquare Adj	0.037816
Root Mean Square Error	1.485149
Mean of Response	6.806752
Observations (or Sum Wgts)	683

Analysis of Variance

Source	DF	Sum of Squares	Mean Square	F Ratio
Model	1	61.3269	61.3269	27.8042
Error	681	1502.0603	2.2057	**Prob > F**
C. Total	682	1563.3872		<.0001*

Parameter Estimates

Term	Estimate	Std Error	t Ratio	Prob>|t|
Intercept	6.8118407	0.056836	119.85	<.0001*
m_PRKCB	-0.959448	0.181956	-5.27	<.0001*
